# Disentangling hindgut metabolism in the American cockroach through single-cell genomics and metatranscriptomics

**DOI:** 10.3389/fmicb.2023.1156809

**Published:** 2023-05-30

**Authors:** Helen E. Dukes, Kara A. Tinker, Elizabeth A. Ottesen

**Affiliations:** ^1^Department of Microbiology, University of Georgia, Athens, GA, United States; ^2^National Energy Technology Laboratory (NETL), Pittsburgh, PA, United States

**Keywords:** cockroach (*Periplaneta americana*), gut microbiome, community genomics, community transcriptomics, intestinal microbiota

## Abstract

Omnivorous cockroaches host a complex hindgut microbiota comprised of insect-specific lineages related to those found in mammalian omnivores. Many of these organisms have few cultured representatives, thereby limiting our ability to infer the functional capabilities of these microbes. Here we present a unique reference set of 96 high-quality single cell-amplified genomes (SAGs) from bacterial and archaeal cockroach gut symbionts. We additionally generated cockroach hindgut metagenomic and metatranscriptomic sequence libraries and mapped them to our SAGs. By combining these datasets, we are able to perform an in-depth phylogenetic and functional analysis to evaluate the abundance and activities of the taxa *in vivo*. Recovered lineages include key genera within *Bacteroidota*, including polysaccharide-degrading taxa from the genera *Bacteroides*, *Dysgonomonas*, and *Parabacteroides*, as well as a group of unclassified insect-associated *Bacteroidales*. We also recovered a phylogenetically diverse set of *Firmicutes* exhibiting a wide range of metabolic capabilities, including—but not limited to—polysaccharide and polypeptide degradation. Other functional groups exhibiting high relative activity in the metatranscriptomic dataset include multiple putative sulfate reducers belonging to families in the *Desulfobacterota* phylum and two groups of methanogenic archaea. Together, this work provides a valuable reference set with new insights into the functional specializations of insect gut symbionts and frames future studies of cockroach hindgut metabolism.

## 1. Introduction

Cockroaches are emerging models for the study of host-diet-microbiota interactions, gut microbiome assembly, and host immune-microbiome interactions (Pérez-Cobas et al., 2015; Mikaelyan et al., 2016; Li et al., 2018). Cockroach symbionts include many taxa commonly found in the human gut microbiome (Eckburg et al., 2005; Lozupone et al., 2012), but with an evolutionary history consistent with an insect gut origin (Schauer et al., 2012). Recent studies have found that the gut microbiota of cockroaches is remarkably stable to dietary perturbations (Pérez-Cobas et al., 2015; Tinker and Ottesen, 2016; Lampert et al., 2019) and has remained highly similar across vast swaths of evolutionary distance (Tinker and Ottesen, 2020). Robust survival and minimal diet requirements of the American cockroach (*Periplaneta americana*) make this organism an attractive lab animal, as does the ability to easily establish germ-free and gnotobiotic animals (Jahnes et al., 2019; Dukes et al., 2021; León et al., 2021).

Like many omnivores, the cockroach hindgut microbiome is dominated by *Bacteroidota* and *Firmicutes*, with *Desulfobacterota*, *Pseudomonadota*, and methanogenic archaea as additional players (Schauer et al., 2012; Dietrich et al., 2014; Schauer et al., 2014; Pérez-Cobas et al., 2015; Mikaelyan et al., 2016; Tinker and Ottesen, 2016; Jahnes and Sabree, 2020). Members of *Bacteroidota* are known as primary fermenters in the guts of both cockroaches and humans: breaking down dietary polysaccharides and host-derived mucins (Salyers et al., 1977; w). *In vitro* and in mice colonized by human gut isolates, this action has been found to liberate useful metabolites for other members of the gut microbiota (Rey et al., 2013; Rakoff-Nahoum et al., 2014), including fermentation end products like H_2_, lactate, and SCFAs (Macy et al., 1978). Cockroach-associated *Firmicutes* are less well-characterized, but studies of organisms from humans and ruminants indicate *Firmicutes* have a variety of functions in the gut, including polysaccharide degradation, reductive acetogenesis, lactate consumption, amino acid fermentation, and SCFA production (Barker, 1981; Rey et al., 2010; Fischbach and Sonnenburg, 2011; Flint et al., 2012; Wang et al., 2020; Karekar et al., 2022). Phylum *Desulfobacterota* contains sulfate-reducing bacteria, who consume H_2_ and other organic compounds (e.g., lactate) as electron donors for the reduction of sulfate and more oxidized sulfur compounds (Rey et al., 2013). Methanogenic archaea utilize H_2_ or formate as an electron donor to reduce CO_2_, methanol, or methylated compounds (e.g., methylamines) to methane in a variety of anaerobic environments (Garcia et al., 2000; Nakamura et al., 2010). While some broad functions of microbial groups have been elucidated as above, more specific activities of lower taxa in the context of this complex gut microbiome are largely unknown.

An obstacle to making meaningful inferences regarding activity and roles of different members of the *P. americana* gut microbiome is their lack of representation in microbial databases (Mikaelyan et al., 2015). As mentioned above, cockroach gut microbes are often found in sister clades to better-characterized mammalian gut microbes. Lack of cultured representatives in these clades limits the extent of functional inferences (Schauer et al., 2012; Mikaelyan et al., 2015). Though some have concentrated on this much-needed task of culturing and sequencing genomes of *P. americana* hindgut microbes (Vera-Ponce De León et al., 2020; Vera-Ponce de Leon et al., 2022), knowledge of the genomic properties of species in this environment remains minimal. A culture-independent understanding of genetic elements present in these microbes and their survival strategies gives insight into microbial mechanisms of stability and resilience in a gut environment.

In single cell-amplified genome (SAG) sequencing, flow cytometry is used to sort individual cells into plates for whole-genome amplification and sequencing. This allows for direct, unambiguous attribution of functions to specific community members, making it a useful tool for functional characterization of uncultured organisms (Kvist et al., 2007; Mußmann et al., 2007; Malmstrom et al., 2013). In contrast, metagenome assembly and binning is highly sensitive to the relative abundance and genome diversity of target groups and can create chimeric assemblies of multiple, closely related genomes (Meyer et al., 2022). A downside of SAGs is that they are often highly fragmented and incomplete, although this can be circumvented to some extent by sequencing of multiple, closely related cells. Nonetheless, our sequenced SAGs generate a culture-independent view of cockroach gut microbe genome content and functional capabilities that capture many characteristics supported in other host systems and provide new insight into possible cockroach-specific functions. To build on these results, we used shotgun metagenomics and metatranscriptomics to evaluate the relative abundance and activity of individual members of the *P. americana* hindgut microbiome *in vivo*.

For each of 96 high-quality SAGs, this work details: phylogeny, transporter complement, carbohydrate breakdown strategies, highly transcribed functions, and relative abundance and activity *in vivo*. We identify multiple groups of organisms with few to no cultured relatives representing potential new species and higher taxa. Some indicate metabolic functions unlike those previously described for their phylum. Using phylogenetically redundant SAGs, we discover activities defining distinct metabolic niches for closely related organisms with similar genomic content. We also note specialty metabolisms that show potential for influencing the activities of other microbes *via* shared products and substrates.

A single-cell understanding of microbial constituents in the context of diverse gut microbiota expands our catalog of extant microbial groups, illustrates niches occupied by community members, and uncovers fine-scale mechanisms of community stability for investigation.

## 2. Materials and methods

### 2.1. Cockroach maintenance and sample collection

Cockroaches are kept on a diet of dog food (Purina One) and water (*via* cellulose sponge) *ad libitum*. They live in a 20-gallon glass fish tank with cube-cut aspen hardwood bedding (TSK Supply), cardboard tubes for housing, and petroleum jelly smeared along the top of the sides of the tank to prevent escape.

An entire gut (foregut to cecum) was dissected out of an adult male *P. americana*, and the hindgut was separated from the midgut and cecum with autoclaved task blades. A fresh autoclaved task blade was then used to slice the hindgut lengthwise, exposing the luminal contents. The sliced hindgut was vortexed in 1 mL 1X phosphate-buffered saline and centrifuged at 2000 × *g* for 30 s to precipitate large debris before 1 mL of supernatant was transferred to a cryovial with 100 μL glyTE (100 mL glycerol, 20 mL 100X TE pH 8, 60 mL deionized water; filter-sterilized and stored at –20°C). This was swirled to mix well, plunged in liquid nitrogen, and kept at –80°C overnight before mailing on dry ice.

### 2.2. Genome sequencing, assembly, and annotation

Cells were sorted and genomes amplified at the Bigelow Laboratory Single Cell Genomics Center (East Boothbay ME) per their protocol (Stepanauskas et al., 2017). For this protocol, a plate of 384 SAGs is initially sequenced at low coverage to evaluate SAG phylogeny and quality. Briefly, individual cells were sorted into wells of a 384-well plate, lysed, and their genomes amplified and used to generate shotgun sequencing libraries. Initial low-coverage sequencing (2 × 100 bp) was performed on an Illumina NextSeq2000 and assembly used SPAdes for the preliminary 384 genomes, and annotation was completed using Prokka (Seemann, 2014; Stepanauskas et al., 2017) with a combination of the bacterial and archaeal Swiss-Prot databases (2017). Preliminary taxonomic assignments were made based on recovered SSU rDNA phylogeny and CheckM (Parks et al., 2015). CheckM also provided initial estimates of genome completeness and contamination by foreign DNA, complemented by tetramer principal component analysis (Woyke et al., 2009).

Following initial quality control, 100 high-quality SAGs of interest were manually selected for deeper sequencing. These final genomes included in our dataset were manually selected from the initial 384 SAGs for high-coverage re-sequencing. Selection was based on estimated contamination and completeness levels, whole-genome amplification Cp values, phylogenetic breadth, and presence/abundance of taxa in hindgut 16S rRNA gene datasets. For these 100, a new pooled library was prepared, sequenced more deeply, and processed with the same bioinformatics pipeline above (Stepanauskas et al., 2017).

Contigs files were used to calculate basic statistics for each genome using CheckM *lineage_wf* (Parks et al., 2015). See full output in Supplementary Table S1-3. Four SAGs exhibiting contamination of 10% or more were excluded from further analyses.

Pairwise average nucleotide identities (ANI) between all genomes were determined using contigs from the SAGs and fastANI (Jain et al., 2018) on default parameters as implemented in the *anvi-compute-genome-similarity* workflow from Anvi’o (Eren et al., 2021). Alignment fraction and percent identity for these comparisons may be found in Supplementary Table S1-7.

Abundance comparisons between SAG taxa in this dataset and in 16S rRNA gene datasets were carried out using sequence data from Tinker and Ottesen (2016). These sequences were processed with DADA2 (Callahan et al., 2016) and classified with SILVA release 138.1. Average relative abundances of phyla, families, and genera were calculated across all diets for comparison with SAGs.

### 2.3. Phylogenetic analysis

Two strategies were used to phylogenetically place SAGs on trees using either 16S rRNA or single-copy marker genes. For 16S rRNA gene trees, near-complete 16S nucleotide sequences from SAGs (931–1,543 bp) were recovered with Metaxa2 and aligned with the SILVA v138.1 SSU Ref NR 99 database (SILVA-NR) (Bengtsson et al., 2011; Quast et al., 2012; Glöckner et al., 2017) using SINA (v1.7.2; Pruesse et al., 2012). Alignments were imported into ARB (v6.0.6; Westram et al., 2004) and filtered to remove positions with majority gaps. SAGs were initially added to the SILVA-NR guide tree using ARB parsimony. Reference species indicated as close relatives based on this initial placement were manually picked from SILVA-NR to build and visualize phylum-specific 16S rRNA gene trees using RAxML (*GTRGAMMA*) (v8.2.12) in ARB (v6.0.6) with rapid bootstrapping (1,000x) (Westram et al., 2004; Stamatakis, 2014).

Separately, genome trees were built for SAGs of the major phyla (*Bacteroidota*, *Firmicutes*, *Desulfobacterota*) by aligning a set of domain-specific, single-copy “marker” genes recovered from the SAG dataset and reference sequences in RefSeq and GenBank (Clark et al., 2016; O’Leary et al., 2016). References were chosen by extracting protein sequences of SAG marker genes with CheckM (*lineage_wf*) and aligning them against the Genome Taxonomy Database (release 95) with DIAMOND (*blastp*) (Buchfink et al., 2015; Parks et al., 2015, 2018, 2020, 2022; Rinke et al., 2021). Hits with a minimum bit score of 50 and identity of 85% (or 80% for *Firmicutes* references) were downloaded from their respective databases. First, we ran CheckM on reference genomes with and without SAGs included to retrieve concatenated marker gene alignments. Both sets of alignments were trimmed with trimAl (option *-gappyout*) (Capella-Gutiérrez et al., 2009). Reference trees were built with RAxML first using alignments of only reference sequences (set parsimony and bootstrap seeds = *12,345, PROTGAMMAWAG*). Reference packages were built from these trees using Taxtastic^1^, and SAGs were placed onto reference trees using pplacer and the trimmed reference-SAG alignments (Matsen et al., 2010). Placement files from pplacer were converted to phyloXML tree files using guppy (*tog*) and visualized in R with package rphyloxml.

For SAGs unable to be placed on either tree due to lack of recovered marker genes (16S rRNA gene or others), PHANTASM was used to find suitable phylogenetic markers and for tree creation (Wirth and Bush, 2023). The gene for valine tRNA-ligase (*valS*) was used to identify references for the tree for SAG D05, the 50S ribosomal protein L19 (*rplS*) was used for SAG F22, and DNA polymerase III (*polC*) for SAG O13.

Organisms were named based on their placement in 16S rRNA gene, genome, and PHANTASM trees. Names and phylum designations used in this paper follow the Genome Taxonomy Database (GTDB) convention (Parks et al., 2018, 2020). Submission of genomes to NCBI required that some taxa be re-named to match the NCBI taxonomy, as specified in w.

Average nucleotide identity was calculated using FastANI (Jain et al., 2018).

### 2.4. Identification of functional genes

Transporters were identified by mapping SAG sequences to the Transporter Classification Database (TCDB) (Saier et al., 2021) using DIAMOND (blastx) (Buchfink et al., 2015). Hits with bit scores greater than 70 were used to characterize transport strategies of SAGs (Supplementary Table S3). TCDB identifiers (TCIDs) are used throughout the text. Transporters were binned at the TCDB family level, and transporter families with a maximum abundance of >6% of all transporters found were included in the stacked bar chart visualization.

Carbohydrate-active enzymes (CAZymes) were annotated with dbCAN standalone version using HMMer (v3.3.2) (Eddy, 2011; Eddy, 2020; Supplementary Table S4). CAZymes associated with polysaccharide-utilizing loci (PULs) were predicted using the PULpy pipeline (Stewart et al., 2018) and databases Pfam (release 33.0) and dbCAN (released April 8, 2020). Absolute CAZyme and PUL counts were normalized by dividing counts in a genome by its total coding sequence length. Because one protein may contain multiple different CAZyme domains, CAZyme transcriptional levels are shown as a heatmap. CAZymes with an average relative abundance >0.5% of a SAG’s transcripts are shown, and columns (SAGs) of the heatmap have been scaled to show levels of CAZymes within each SAG.

Metabolic heat maps for KEGG orthologs (KOs) were created by first identifying KOs in the SAGs using KofamScan (Kanehisa, 2000; Kanehisa, 2019; Aramaki et al., 2020; Kanehisa et al., 2021). KOs were then mapped to transcriptional data from each SAG, and functions were binned at hierarchical BRITE levels. Visualization uses KEGG categories at the third level of BRITE hierarchy. Pathways displayed were manually chosen, and proportions of SAG transcripts corresponding to KOs were column scaled to visualize relative activity of KOs within each SAG.

Enzymes involved in Dsr-dependent dissimilatory sulfur metabolism were identified using DiSCo (Neukirchen and Sousa, 2021).

Single cell-amplified genomes amino acid files were used to search for evidence of bacterial microcompartments (BMCs). These were fed into BMC Caller to search for shell proteins (Sutter and Kerfeld, 2022). Loci with shell proteins were tested for locus type using BMC Caller online.

Searches for genes encoding other specialized metabolic capabilities relevant to gut microbiome lifestyle were carried out using HMMer v.3.3.2 (*hmmsearch*). See Supplementary File S1 for details on downloading alignments and HMM profiles for these genes. HMM profiles were built from alignments using HMMer v.3.3.2 (*hmmbuild*), and hits to HMM profiles were determined using *hmmsearch* with family-appropriate cutoffs (Eddy, 2020).

### 2.5. Metagenomic and metatranscriptomic mapping

We mapped 25 metagenome and 20 metatranscriptome libraries to our SAGs to get information regarding typical abundance and activities across a range of conditions. See Supplementary File S1 for information on sample preparation, DNA extraction, library preparation, and sequence processing for metagenome and metatranscriptome datasets.

Processed metagenome and metatranscriptome sequences were separately mapped to the SAG dataset and RefSeq Microbial Genomes (O’Leary et al., 2016) using DIAMOND v2.0.9 (Buchfink et al., 2015) (blastp --sensitive --top 1 --minscore 50). Bit scores of all hits with *e*-value <10^−10^ were compared across the SAG and RefSeq databases to assign hits to the best match from any source. To calculate taxon abundance, reads with equal top hits to multiple SAGs were counted as a hit to each SAG. Note that this can over-count groups of closely related SAGs and highly conserved genes. To calculate transcriptional abundance within a SAG, transcripts were first extracted by SAG, then—for equivalent top hits within a single SAG—the hit was given to the SAG sequence with more hits. As we have groups of SAGs displaying high ANI, we preferred to over-count conserved genes and SAG abundance than under-count SAGs in closely related groups.

**Figure 1:**
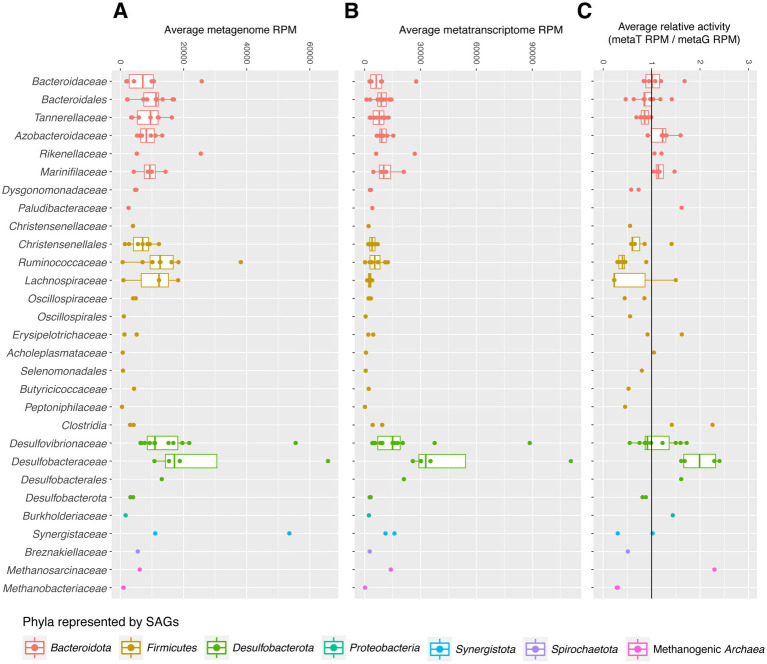
**(A–C)** SAG reads per million (RPM) in metagenome **(A)** and metatranscriptome **(B)** datasets were used to visualize relative abundance. The relative activities of SAGs **(C)** were measured by dividing average RPM of SAGs’ transcripts in the metatranscriptome dataset **(B)** by their RPM in the metagenome dataset **(A)**.

Relative transcriptional activities of SAGs were calculated for high abundance families by dividing the average reads mapped per million (RPM) of the SAG in the metatranscriptome dataset by its RPM in the metagenome dataset. RPM (often TPM) is commonly used to summarize transcript abundance normalized by gene length; here, we use it to normalize SAG abundance by genome assembly length. For each SAG, average RPM across all samples is shown. Reads per kilobase (RPK) was first calculated by dividing the number of reads in each sample (metagenome or metatranscriptome) mapped to a SAG by the total coding sequence length of that SAG to account for differences in genome completeness. The sum of all SAGs’ RPKs in each sample was divided by 1,000,000 to produce a normalization factor for sample sequencing library depth. For each sample, the SAG’s RPKs were divided by the sample library depth normalization factor, and the average RPK across all samples for each SAG is binned by family, as shown in Figures 1A–C.

## 3. Results and discussion

### 3.1. Dataset-wide statistics

One hundred single cell-amplified genomes (SAGs) were recovered with 42/100 genomes estimated as at least 50% complete by CheckM. Over half of the genomes (59/100) indicate 0% contamination, and 96/100 exhibit less than 10% contamination. The four genomes with contamination over 10% were excluded from further analyses and are not included in Table 1 statistics (Supplementary Table S1-3).

**Table 1:** Summary of SAG statistics from CheckM (*lineage_workflow*).

	Average	Minimum	Maximum
Number of raw reads	3,530,392	2,976,414	5,272,988
Assembly length (bp)	1,496,472	323,329	3,324,496
Number of contigs	127.7	34	318
Max contig length (bp)	80,627	12,518	267,216
Total coding sequence length (bp)	3,371,229	521,551	13,011,140
Number of predicted genes	1,388	316	3,232
CheckM completeness estimate (%)	47.8	10.3	86.0
GC content (%)	46	27	59
Contamination (%)	0.513	0.00	9.82

Single cell-amplified genomes (SAGs) with contamination estimates >10% are not included in the calculation of the statistics. View Supplementary Table S1-3 for per-SAG data breakdown.

**Figure 2:**
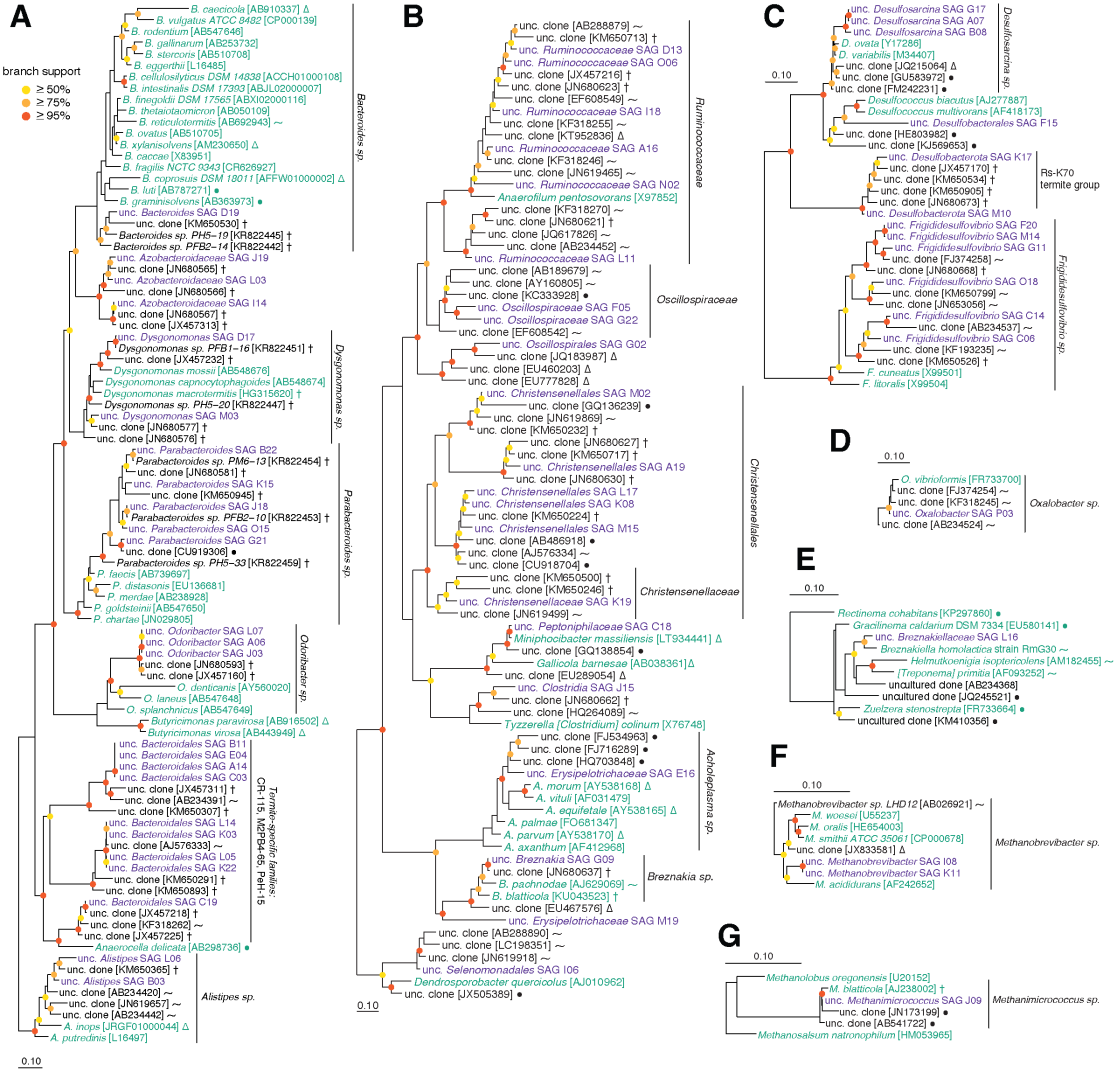
**(A–G)** Phylogenetic trees built using aligned 16S rRNA gene sequences from this SAG dataset and SILVA-NR reference database. Sequences were aligned with SINA, and 16S rRNA gene alignments were filtered to remove positions with majority gaps, resulting alignment lengths described below. Trees were constructed with RAxML and represent the following taxa (followed by alignment length): **(A)** *Bacteroidota* (1,481 bp), **(B)** *Firmicutes* (1,433 bp), **(C)** *Desulfobacterota* (1,511 bp), **(D)** *Proteobacteria* (1,511 bp), **(E)** *Spirochaetota* (1,475 bp), **(F)** *Methanobacteriota* (1,454 bp), **(G)** *Halobacteriota* (1,454 bp). Purple sequences are from SAGs in this dataset, green are from type strains, and black are other references from SILVA. Symbols for references recovered from environmental sources: *P. americana* and other cockroaches (†), termites and other insects (⁓), non-insect animals (∆), non-host-associated sources (●). SILVA accessions are found in brackets. Branch support values above 50% are displayed using colored circles (1,000 bootstraps): red ≥95%, orange ≥75%, yellow ≥50%.

Single cell-amplified genomes were manually assigned to taxonomic groups based on phylogenetic analyses of 16S rRNA genes, as well as genome phylogeny using a concatenated alignment of single-copy marker genes identified by CheckM. We recovered 16S rRNA gene sequences from 83/96 SAGs (Figure 2), and 84/89 SAGs from the three main phyla (*Bacteroidota*, *Firmicutes*, *Desulfobacterota*) included single-copy genes that could be used to further guide their placement (Parks et al., 2015). Three SAGs were not able to be placed on either 16S rRNA gene or marker gene trees. Using other conserved genes as described in Methods, these organisms were tentatively identified as members of *Synergistaceae* (D05), *Clostridia* (F22) and *Ruminococcaceae* (O13) (Supplementary File S1). Although most 16S rRNA gene neighbor references from SILVA-NR are microbes isolated from closely related host species (i.e., cockroaches, termites), some taxa were more closely related to mammalian or even non-host-associated sequences.

Single cell-amplified genomes were found to belong to the following bacterial phyla: *Bacteroidota* (43% of SAGs), *Firmicutes* (31%), *Desulfobacterota* (19%), *Pseudomonadota* (1.0%), *Spirochaetota* (1.0%), and *Synergistota* (2.1%). Three SAGs were identified as Archaea (2.1% from *Methanobacteriota*, 1.0% from *Halobacteriota*). Phyla identified in the 16S rRNA gene dataset that are not found in the SAG dataset are primarily lower-abundance taxa, which include members of *Verrucomicrobiota* and archaeal phylum *Thermoplasmatota* (typically present at 1–2% of 16S rRNA gene libraries).

Within found phyla, most families abundant in 16S rRNA gene data are also represented here (Supplementary Tables S1-5); notable exceptions are *Firmicutes* families *Enterococcaceae* and *Lactobacillaceae* (3.5 and 4.7% in 16S rRNA gene libraries, respectively). CheckM taxonomic analysis of the 386 low-coverage genomes found no SAGs classified as *Lactobacillales* even in this broader sampling, suggesting a methodological bias against these groups. One source of bias may come from sample preparation for SAG sequencing. Vortexing after slicing open may not liberate microbes in strong association with the gut wall. Similarly, cell-sorting may bias against filamentous organisms, which includes many clostridial species (Hedblom et al., 2018). However, 16S sequencing also presents bias, as some taxa are known to carry multiple copies of 16S rRNA genes (Větrovský and Baldrian, 2013), and even “universal” primers do not amplify all organisms with equal efficiency.

To measure the diversity of sampled SAGs, pairwise average nucleotide identity (ANI) was calculated between all genomes. ANI values and tree placement indicate we have a diverse collection of SAGs: with two thirds lacking significant ANI (80%) with any other SAG. However, one third of SAGs (32/96) exhibit 80–100% ANI with at least one other SAG, and 17 of those share >99% ANI with a match, suggesting recovery of multiple isolates of the sample species (Ciufo et al., 2018).

To better evaluate the *in vitro* abundance and activities of organisms captured in our SAG library, we mapped metagenomic and metatranscriptomic reads from the hindguts of cockroaches fed a variety of diets to our SAGs and to RefSeq. 38.4–73.1% of metatranscriptome (mean 62.3%) and 18.7–74.7% of metagenome reads (mean 49.0%) matched either RefSeq or this SAG dataset. 14.2–64.7% of metatranscriptome reads with hits (mean 39.4%), and 22.6–49.0% of metagenome reads (mean 38.1%) mapped with equal or higher bit scores to our SAGs than to any other bacterial/archaeal genome currently present in RefSeq (see Supplementary Tables S1-9, S1-11 for details). Average transcriptional activities of SAGs—normalized by their average abundance in the metagenome dataset—are displayed in Figure 1. Most SAGs have comparable abundances in the metatranscriptome and metagenome datasets, as seen by their placement near 1 in Figure 1C. However, other groups do show higher average transcriptional activities, with *Methanosarcinaceae* and *Desulfobacteraceae* showing some of the highest transcriptional activities. Many *Firmicutes* showed lower relative activity levels, although *Clostridiaceae* SAGs are associated with higher relative activity levels than most in its phylum.

**Figure 3:**
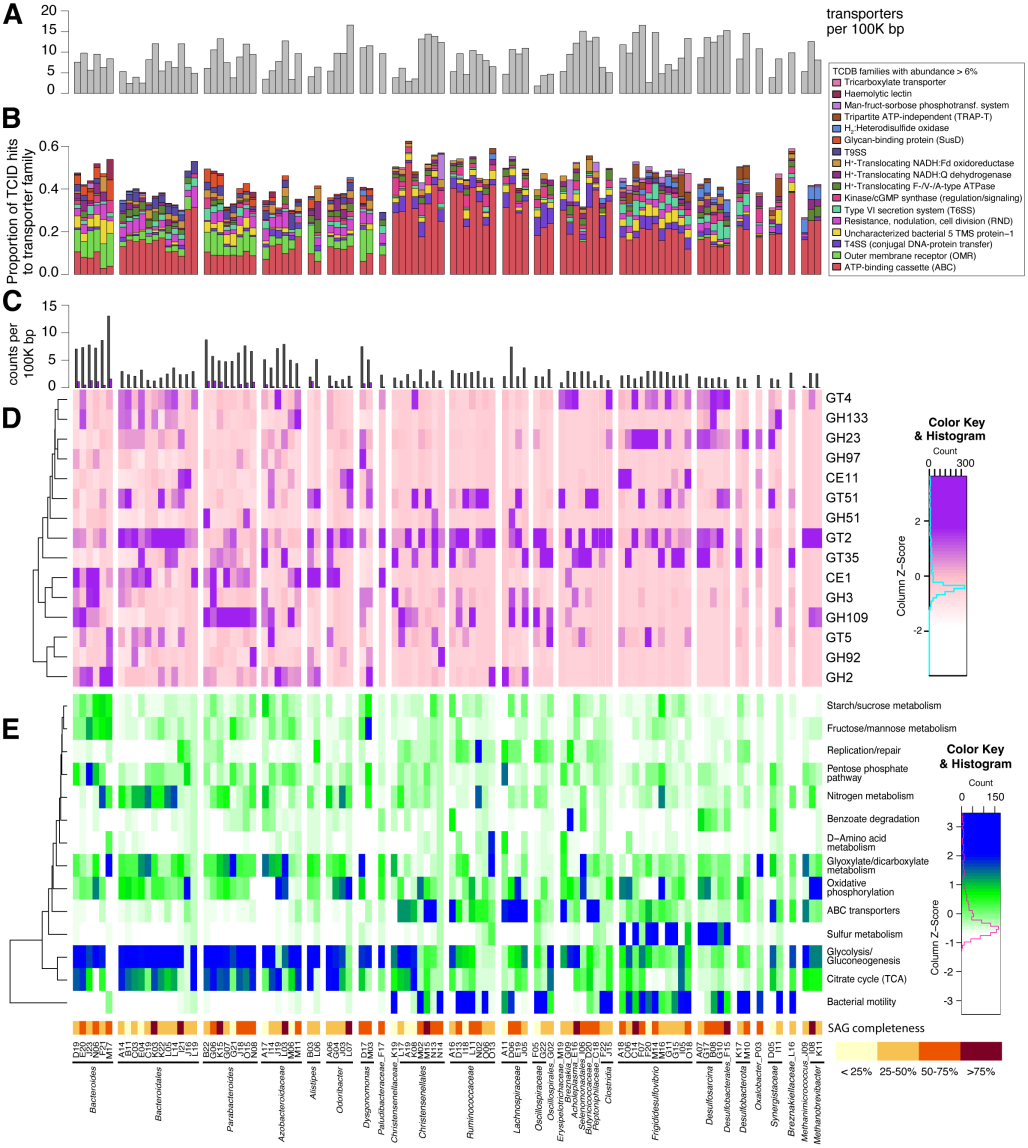
**(A–E)** Histogram of transporters found in SAG genomes **(A)** and stacked bar chart of associated transporter families matching sequences **(B)**. Transporter families with a maximum abundance of at least 6% of transporter-related genes within SAGs are listed at right. Histogram in **(C)** shows counts of CAZymes and polysaccharide-utilizing loci (PULs in purple) found with PULpy and HMMer, normalized by genome size. Part **(D)** displays relative transcriptional abundance of CAZy domains. CAZymes with a maximum proportion of at least 0.5% of transcripts from SAGs are shown here. This relative abundance is scaled by columns to highlight transcriptionally abundant domains within SAGs. Rows are clustered based on CAZyme transcriptional patterns. Heatmap of KEGG pathways represented by SAG transcripts is shown in **(E)**. Metatranscriptome reads from SAGs were mapped to KEGG orthologs (KOs), and KEGG pathway abundance was calculated using its proportion of mapped reads within each SAG. *Z*-scores are scaled by columns to emphasize differences in KEGG pathway transcriptional abundance between SAGs. Pathways included in this figure were manually chosen. Rows are clustered based on KO transcriptional patterns.

Many of the most abundant KEGG functions associated with our SAGs include key housekeeping activities such as protein production (sigma factors, RNA polymerase, elongation/termination factors, non-specific chaperones, quality control peptidase/RNases) and carbon flux maintenance (glycolysis, TCA cycle, and related enzymes). Other key functions include oxygen radical detoxification (superoxide dismutase, peroxiredoxins) and iron sequestering (bacterioferritin). Highly abundant orthologs for glycolysis and the TCA cycle are especially common in *Bacteroidota* SAGs (Figure 3E). Members of *Frigididesulfovibrio* and *Desulfosarcina* indicate an abundance of sulfur metabolism transcripts. *Lachnospiraceae* and *Christensenellales* SAGs transcribe high amounts of ABC transporters. We also notice variability in the transcriptional levels of KEGG orthologs within taxonomic groups that may be caused by incomplete genome recovery. For instance, sulfate adenylyltransferase (*sat*; K00958) and dissimilatory sulfite reductase (*dsrAB*; K11180/K11181) are sulfate metabolism genes with some of the highest average transcription levels across all *Frigididesulfovibrio*. However, *Frigididesulfovibrio* SAGs with low transcriptional activity of sulfur metabolism (C06, F07, I05, and M16) are all missing *dsrAB* from their genomes, in addition to *sat* missing from C06, F07, and M16. Similarly, *Christensenellales* SAGs transcribing more glycolysis orthologs and fewer ABC transporters (K19, L17, A19, and K08) are less complete (Figure 3E) and have fewer instances of ABC transporters present in the genome. Therefore, some of the within-taxon transcriptional variability seen in Figure 3E is likely an artifact of the genes recovered in each SAG. Information on genome completeness is available in Figure 3E and Supplementary Table S1-3, and information on KEGG ortholog recovery for each genome is available in Supplementary Table S2-2.

We were able to identify distinct patterns in the type and proportion of transporter families and CAZymes present across clades. We analyzed the presence/absence and transcriptional abundance of SAG genes associated with key symbiotic functions, including transporters, carbohydrate-active enzymes, genes for sulfate reduction, methanogenesis, and bacterial microcompartments (see Supplementary methods and Supplementary File S1 for full list). Given that uric acid is a form of nitrogen storage in the cockroach fat body, we also probed SAGs for genes involved in uric acid metabolism. However, we found no genes matching uricase (pucL; HMM domain score > 168.4) in any SAGs. Proteins above the HMM threshold for urease (urea amidohydrolase, ureC; HMM domain score > 680.0) were also not found, although many SAGs contained proteins annotated as amidohydrolases. Possible nitrogen-fixing bacteria present in *P. americana* include *Paludibacter*, *Bacteroides*, and *Dysgonomonas* species (Inoue et al., 2015), but nitrogenase (NifH) was only found in methanogen SAGs. Genomic elements were combined with transcriptional profiles to learn active key metabolisms for single cells in this environment.

The top five transporters found in our SAGs include histidine kinases, SusC, type VI secretion systems (T6SS), putative DNA transformation systems, and outer membrane receptors (OMR). SAG taxa showed strong differences in TCDB family abundance within their genomes (Figure 3B). Outer membrane receptor/TonB-dependent receptor family proteins were strongly associated with the *Bacteroidota* phylum. These types of transporters are present in all free-living *Bacteroidota* and promote their canonical polysaccharide-degrading niche, which is commonly studied in human gut isolates (Martens et al., 2008). *Desulfobacterota* and methanogenic Archaea contain the greatest proportion of hydrogen:heterodisulfide oxidoreductases (likely representing Dsr and HdrABC, respectively). *Desulfobacterota* also has the greatest proportion of T6SSs. A T6SS strategy common in SAGs of all taxa reported here (TCID 3.A.23.6.1) may confer escape from host cells (Böck et al., 2017).

Carbohydrate-active enzymes (CAZymes) are identified in all SAGs, ranging from two to 188 per genome. The average number of CAZymes is 40.4 per genome or 3.98 per 100,000 bp sequenced. We find 248 unique CAZyme families in this dataset, including 18 carbohydrate binding modules (CBMs) and 142 glycoside hydrolases (GHs). Figure 3D shows transcriptional abundance of 15 CAZyme families with maximum relative abundance >0.5% in at least one SAG’s transcripts. One of the most widespread and highly transcribed CAZyme domains across our dataset is the GT2 family, but the wide range of specificities in this family limits inferences that can be made. Human gut methanogens are thought to use this domain to produce *N*-acetylneuraminic acid (Neu5Ac)—a predominant sialic acid found on human mucus/epithelial surfaces—to mimic host gut glycans (Samuel et al., 2007). This suggests a potentially important role for this domain in our SAGs’ interaction with the cockroach host.

Cellulosome components were also found (HMM domain score > 24.1). HMMer identified cohesin (PF00963) in *Christensenellales* M02. Dockerins (PF00404; HMM domain score > 22.9) were found in *Alistipes* B03, *Bacteroides* D19/J23, *Bacteroidales* M11, *Acholeplasma* E16, and *Christensenellales* L17/M02/M15/N14, and match scores were highest in *Firmicutes* SAGs. Interestingly, dockerin genes identified by HMMer are assigned to a KEGG entry for stage-II sporulation protein (K06381). Other functions that are more specific to individual bacterial groups are discussed in their respective sections below.

We also examined SAGs for the presence of genes encoding bacterial microcompartments (BMCs). BMCs are metabolic organelles composed of a protein shell that functions to localize substrates and enzymes (Sutter et al., 2021). Evidence of microcompartment shell proteins is found in six SAGs. These organisms are all classified as either *Desulfobacterota* or *Lachnospiraceae*. One *Desulfobacterota* (M10) and one *Lachnospiraceae* (J05) were found to have complete microcompartment loci (containing all genes necessary: shell proteins, regulator, aldehyde dehydrogenase, PTAC, alcohol dehydrogenase, signature enzyme). BMCs identified are estimated to participate in metabolism of ethanolamine, fucose/rhamnose, choline, and taurine (Sutter and Kerfeld, 2022).

**Figure 4:**
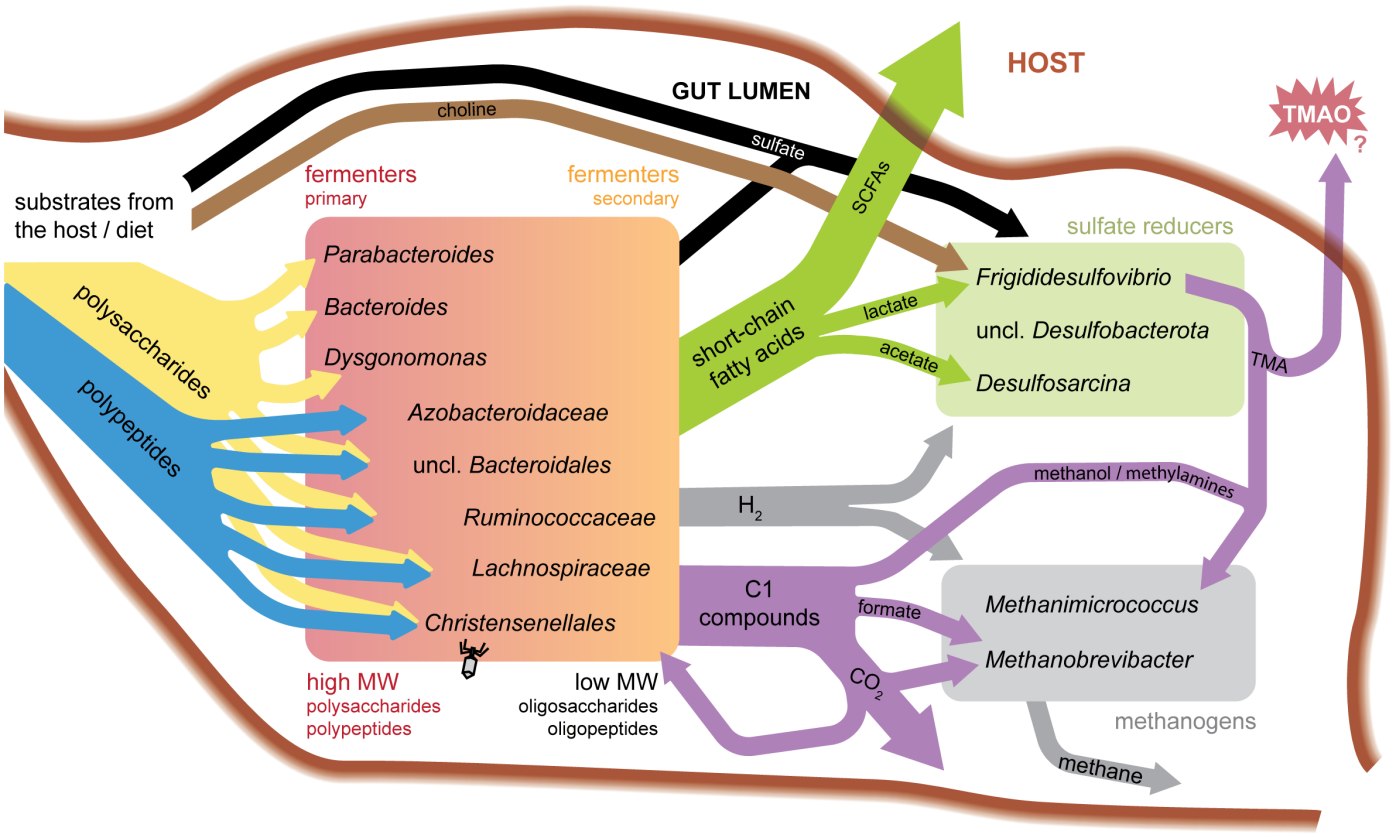
Model of substrate flow in the cockroach hindgut.

Together, these data provide new insight into potential functional activities of a wide diversity of cockroach gut microbiota, as summarized in Figure 4. Below, we discuss specific findings for each of the major sub-groups. We focus on nutrient partitioning between groups of microbes sharing similar functional activities and their potential to impact their peers and host.

### 3.2. Bacteroidota

All SAGs classified as *Bacteroidota* were placed in the *Bacteroidales* order, with the most common genera being *Parabacteroides, Bacteroides, Odoribacter, Dysgonomonas*, and *Alistipes*. These genera are all within the top 10 most abundant of all genera found in *P. americana* 16S datasets (Supplementary Table S1-6). The family *Azobacteroidaceae* is also relatively common, with six representatives that could not be assigned to a genus. This family does not exist in SILVA and therefore its 16S prevalence cannot be compared. *Bacteroidota* SAGs are assigned to 27 putative species-level clusters based on ANI, including an *Odoribacter* cluster comprising four SAGS (A06, G04, J03, L07). Some SAGs may represent clones of the same genome based on tree placement and 97–99% ANI over aligned fragments (Supplementary Table S1-7), while others indicate more remote relationships. Recovering multiple representatives from the same taxon allows us to look for shared functions in the members of that group.

Based on 16S rRNA gene phylogeny, cockroach SAGs classified as *Bacteroides* spp. belong to a single clade distinct from most *Bacteroides* type strains in SILVA (Figure 2A). Similarly, phylogenomic analysis (Supplementary File S1) clusters our SAGs with other *Bacteroides* isolates from *P. americana* (Vera-Ponce De León et al., 2020). This is consistent with previous studies showing distinct insect-gut and mammalian-gut adapted lineages of *Bacteroidota* as well as evidence of phylosymbiosis (Schauer et al., 2012w Tinker and Ottesen, 2020). Several diverse SAGs are placed within the genus *Parabacteroides*. Seven of the eight *Parabacteroides* (B22, G06, G07, G21, J18, K15, O15) show low average intra-genus ANI (89.2%), supported by variable 16S rRNA gene tree placement. Many of their closest relatives were also isolated from *P. americana* and other cockroaches, although one SAG (G21) shares more similarity with a bacterium isolated from an anaerobic wastewater digester, potentially indicating more widespread environmental distribution. The six *Azobacteroidaceae* SAGs form a monophyletic group that shares ANI <80%. Only two well-characterized candidate genera are found in GTDB for *Azobacteroidaceae*: *Azobacteroides* and *Symbiothrix*. We note that the G + C content of our *Azobacteroidaceae* SAGs is substantially higher (40.2% average) than *Azobacteroides pseudotrichonymphae* (30.1% average), suggesting considerable evolutionary distance from this microbe. However, both genera have members that are symbionts of termite gut protists (endo- and ecto-, ciliates and flagellates) (Hongoh et al., 2007, 2008), so it is possible our *Azobacteroidaceae* SAGs represent another clade of *Azobacteroidaceae* that interacts with cockroach protists. Presence of protists in our stock cockroaches has been confirmed *via* light microscopy (data not shown), although their identity has not been established.

Twelve *Bacteroidales* SAGs fall within a monophyletic group that represents a poorly characterized, insect-derived lineage (Figure 2A). In both 16S rRNA gene and genome trees, these *Bacteroidales* SAGs are placed into groups populated only by cockroach and termite references (Figure 2A; Supplementary File S1). We identify two distinct clades, each with four SAGs (A14-B11-C03-E04 and K03-K22-L05-L14), that share high within-clade ANI values (averaging >99%) but no between-clade overlap. A single 16S rRNA gene reference sequence for one of these clades is included in DictDB (Mikaelyan et al., 2015), where it is classified as a member of *Porphyromonadaceae* Gut Group Termite Cluster I. In SILVA, they fall into the PeH15 and M2PB4–65 termite groups. As the taxonomy of these specialty families is poorly developed, we have designated these strains as unclassified *Bacteroidales*.

Carbohydrate-active enzymes (CAZymes) are significantly more common in *Bacteroidota* SAGs than any other phylum (*p* ≤ 0.01, one-tailed *T*-test), with an average of 5.28 CAZymes per 100,000 bp sequenced. We identified 191 different CAZymes in this group. *Bacteroidota* is the only phylum of this dataset containing polysaccharide-utilization loci (PULs), of which we identified 229 total. Approximately half of all PULs found include an associated CAZyme (111/229). While this is lower than a previous study of cockroach *Bacteroidota*, which found approximately 75% of PULs with CAZymes (Vera-Ponce De León et al., 2020), this is likely due to the fragmentary nature of our SAGs (average number of contigs per SAG = 128; average contig size = 12.5 Kbp).

Based on RNA:DNA ratios, members of *Bacteroidota* are moderately active on average, with different families showing metatranscriptome abundance levels higher, lower, or equal to their abundance in the metagenome (Figure 1C). Highly expressed transcripts include many of the transport and carbohydrate-degrading genes described above. This is consistent with the long-standing association between *Bacteroidota* and primary degradation of complex polysaccharides in gut environments (Figure 4). However, activities of different groups within this phylum demonstrates that these groups each fulfill unique niches within the gut environment.

#### 3.2.1. Bacteroides and Parabacteroides

Six *Bacteroides* and eight *Parabacteroides* SAGs were recovered. These closely related genera show substantial overlap in functional capacity and transcriptional activity. All *Bacteroides* and *Parabacteroides* SAGs contain both CAZymes and PULs. At least some components of all PULs in *Bacteroides* and *Parabacteroides* SAGs are transcribed, with SusCD often the highest transcribed components.

The highest transcribed PUL in *Bacteroides* D19 indicates activity on xylan, given its domains with xylosidase/arabinofuranosidase (GH43, GH146) and xylanase (GH10, GH5_21) functions. Common CAZymes found in PULs of this dataset (CE1, GH2, GH3, and GH43) can work together to degrade polysaccharides often found in wood/cereal grains. CE1 functions to break carbohydrate polymer backbones into their constituent sugars. Family GH43 domains remove arabinofuranose groups from an arabinoxylan backbone, and GH2/GH3 have glucosidase activity.

Though *Bacteroides* and *Parabacteroides* SAGs show similar transporter and polysaccharide strategies in their genomes, we see distinct CAZyme activities for these genera in their transcripts (Figure 3D). CAZymes transcribed by *Bacteroides* SAGs are diverse, as shown in Figure 3D—including CE1, GT2, GH2, GH3, and GH109. Furthermore, the top five CAZymes from any one *Bacteroides* SAG are seldom shared with other SAGs from its taxon. In contrast, *Parabacteroides* SAGs appear more specialized, as GH109 (α-*N*-acetylgalactosaminidase) is consistently the single top transcribed CAZyme from this group.

Results in Figure 3D are similar to (Vera-Ponce De León et al., 2020), in which researchers found cultured *Bacteroides* from the cockroach gut targeted a higher diversity of substrates than *Parabacteroides*. GH109 is a domain found to catabolize mucin-type *O*-glycans (Liu et al., 2007), while CE1, GT2, GH2, and GH3 have broad substrate specificities (Harvey et al., 2000; Breton et al., 2005; Talens-Perales et al., 2016; Nakamura et al., 2017). Prioritizing GH109 may allow *Parabacteroides* to feed off host glycans, rather than competing with *Bacteroides* for high-demand substrates in the cockroach gut. *Bacteroides* species meanwhile may focus on diverse dietary polysaccharides. These complementary primary fermentation functions reflect overall ecological actions of *Bacteroidota* found in host systems (Salyers et al., 1977; Rey et al., 2013; Vera-Ponce De León et al., 2020).

One surprising feature is that both genera show high transcriptional activity of proteins annotated as RND efflux pumps (TCID 2.A.6), indicating this is a key function in these organisms. This is interesting, as the most well-established function for RND efflux pumps in the human gut isolate *Bacteroides fragilis* is antibiotic resistance (Ueda et al., 2005). As our cockroaches have not been exposed to exogenous antibiotics, this suggests that RND efflux pumps serve a broadly important function in the cockroach gut, likely eliminating antibiotics or toxins produced by other microbes or the host (Fernando and Kumar, 2013).

#### 3.2.2. Dysgonomonas

Two *Dysgonomonas* SAGs were recovered, both containing PULs at a density close to that of *Bacteroides* and *Parabacteroides* SAGs and transcribing a variety of CAZymes (Figure 3). Like *Bacteroides* SAGs, orthologs in fructose/mannose and starch/sucrose metabolisms show significant transcriptional activity in *Dysgonomonas* SAGs (Figure 3E). *Dysgonomonas* D17 also transcribes an uptake-hydrogenase (3.D.7.2.3), which is used in hydrogen production from sugar fermentation (Zbell and Maier, 2009). Hydrogen is a substrate capable of cross-feeding with sulfate reducers and methanogens. These activities support polysaccharolytic function of our *Dysgonomonas* SAGs.

#### 3.2.3. Azobacteroidaceae

The six *Azobacteroidaceae* SAGs have genomic CAZyme density similar to *Bacteroides* and *Parabacteroides* SAGs, but *Azobacteroidaceae* transcripts for starch-utilization systems and polysaccharide metabolism are not as common as in the other taxa. *Azobacteroidaceae* M11 only transcribes two of its five PULs, and PULs are less common in this family than *Bacteroides* and *Parabacteroides* (Figure 3C). SAGs in this family also do not show a consistent transcriptional CAZyme pattern (Figure 3D). *Azobacteroidaceae* in this dataset display prevalent transcripts involved in glyoxylate and dicarboxylate metabolism (Figure 3E), especially converting propionyl-CoA to succinyl-CoA.

The closest reference, *Symbiothrix dinenymphae*, exhibits both amino acid and cellulolytic abilities in relationship with its termite protist host (Yuki et al., 2015). Our SAGs do not indicate specialization in cellulose breakdown, however propionyl-CoA is produced by branched-chain amino acid and odd-chain fatty acid catabolism. This and their relatively high level of nitrogen metabolism orthologs (Figure 3E) suggests they may be metabolizing amino acids, either individually or in association with a protist host.

#### 3.2.4. Unclassified *Bacteroidales*

Twelve SAGs (A14, B11, C03, C19, E04, I21, J16, K03, K22, L05, L14, L19) belong to a monophyletic group of unclassified *Bacteroidales*. These genomes show a lower proportion of OMR transporters and fewer CAZymes than other taxa in this phylum (Figure 3C). Most *Bacteroidota* SAGs lacking PULs are unclassified *Bacteroidales*. CAZyme domain GT2 shows high transcriptional activity (Figure 3). As mentioned above, GT2-containing proteins have several potential activities, but methanogens of the human gut may use them to mimic host gut glycans (Samuel et al., 2007). Other host-association proteins such as adhesin molecules (cadherin-like) and immune system modulators (hemagglutinin, gingipain, streptopain, C5a-peptidase, K09607) are also transcribed in these SAGs.

Top transcripts from three of these *Bacteroidales* SAGs (K03, L05, L14) are gliding motility-associated proteins (K20276, BapA), T9SSs, and the Sec proteins required by T9SSs (TCID 2.A.130) for effector protein export (Mcbride, 2019). With host-like glycans produced *via* GT2, other microbes may even adhere to this bacterium and be pulled along *via* its gliding motility, as in another *Bacteroidota* member of the oral microbiome (Shrivastava et al., 2018). Indeed, BapA (K20276), has been shown to promote biofilm formation in a diversity of bacteria, and has been implicated to mediate cell–cell interactions (Latasa et al., 2005).

Although many top transcripts mapping to this group encode proteins of unknown function, it is clear these unclassified *Bacteroidales* have a distinct functional profile from other *Bacteroidota*, potentially related to host–microbe or microbe–microbe interactions rather than primary fermentation of polysaccharides.

### 3.3. Firmicutes

We identify a high diversity of *Firmicutes* members in our SAG dataset. *Firmicutes* SAGs are placed into the following families: *Ruminococcaceae*, *Christensenellaceae*, *Lachnospiraceae*, *Oscillospiraceae*, *Erysipelotrichaceae*, *Butyricicoccaceae*, *Peptostreptococcaceae*, and *Acholeplasmataceae* (Figure 2). From *Bacteroidota*, we recovered multiple members of a few select genera, but *Firmicutes* SAGs show a high degree of genomic diversity with little overlap between recovered genomes. *Ruminococcaceae* SAGs are the least diverse, encompassing a cluster of six related genomes and a member of a sister clade (L11). Two of these (D13 and A11) are the only two *Firmicutes* that align sufficiently to allow ANI calculation, although similarity was relatively low (85.1% over 16% of D13 genome; Supplementary Table S1-7). The seven *Christensenellales* SAGs are not placed into any currently described family and are instead found in 2–3 clades basal to *Christensenellaceae*. Similarly, one SAG from *Oscillospirales* is placed basal to *Oscillospiraceae* near 16S rRNA gene sequences recovered from mammalian feces. The *Lachnospiraceae* SAGs include one *Breznakia* organism and three additional SAGs, each belonging to separate clades of this family. Two *Firmicutes* SAGs are classified at the genus level: *Acholeplasma* E16 and *Breznakia* G09. With little phylogenetic overlap, analyses of *Firmicutes* SAGs indicate a diverse collection of organisms with few closely related relatives in reference databases.

Three SAGs could be classified by their 16S rRNA gene but did not have closely related genomes in RefSeq. *Selenomonadales* I06 shows 16S rRNA gene similarity with *Dendrosporobacter* spp., but searching RefSeq using marker genes from this SAG did not identify *Dendrosporobacter quercicolus* as a reference for the genome tree. Furthermore, while the closest relatives by 16S rRNA gene analysis of *Oscillospirales* G02 and *Acholeplasma* E16 are uncultured clones from mammals, they lack close relatives with sequenced genomes in RefSeq (Supplementary File S1). This illustrates that, although there is evidence of SAGs from these taxa in similar environments, the genomes in this dataset represent largely unknown groups.

Polysaccharide degradation and production of SCFAs are often the focus of gut *Firmicutes* discussions in the rumen and human gut (Barcenilla et al., 2000; Cockburn and Koropatkin, 2016). However, we find fewer CAZyme domains per bp of genome in *Firmicutes* than *Bacteroidota* SAGs (value of *p* < 0.01). Of the CAZymes present, GT2 is highly transcribed in many *Firmicutes* SAGs (polyspecific, polysaccharide synthesis including glycans and cellulose). GT51 (peptidoglycan glycosyltransferase), GH109 (mucin-type *O*-glycan metabolism), and GT35 (glycogen/starch phosphorylase) are variably transcribed across taxa and do not show group-specific patterns. Dockerins for cellulosomes are present in genomes of *Firmicutes* SAGs, especially members of *Christensenellales*. However, the transcriptional levels of these are quite low. Although we find polysaccharolytic evidence in top transcripts by some *Firmicutes* SAGs (e.g., *Ruminococcaceae* L11, *Oscillospiraceae* G22), activities of our organisms instead support their utilization of a diversified set of metabolic substrates.

To facilitate their metabolic endeavors, *Firmicutes* transcribe many ABC-transport systems (Figure 3E). This includes transporting branched-chain amino acids (K01997-K01999), peptides (TCIDs 3.A.1.5), and polysaccharide constituent sugars (TCIDs 3.A.1.1) (Chow et al., 2007). *Firmicutes* SAGs in many cases show high transcription of various pathways for utilization of these amino acids, sugars, and—in some cases—a potential for autotrophic CO_2_ reduction. This is consistent with reports that this phylum can engage in amino acid fermentation and carbon fixation in humans (Amaretti et al., 2019; Guo et al., 2020; Trischler et al., 2022). It also supports *Firmicutes* as secondary degraders of polysaccharides, as demonstrated in simplified co-cultures of human isolates (Falony et al., 2006). Finally, prevalent transcription of motility-related genes unites *Firmicutes* SAGs (Figure 3E).

*Firmicutes* SAGs also show high potential for genetic exchange. This is in agreement with a study of mobile genetic elements in the human gut (Jiang et al., 2019). Type IV secretion systems (T4SSs) functioning in transformation, conjugation, or other putative protein/DNA secretion systems are prevalent in our SAGs and actively transcribed by these groups (Figure 3B), as are transposases (K07497, K07486) and recombinases (K06400). Of 108 instances of relaxase in our SAGs, 54 belonged to *Firmicutes* genomes, while *Bacteroidota* and *Desulfobacterota* split most of the remaining (25 and 26 respectively). This suggests this group may have a higher degree of genomic plasticity than many in the gut. In accordance with this, our *Firmicutes* SAGs exhibit low genomic overlap combined with high phylogenetic and functional diversity.

#### 3.3.1. Ruminococcaceae

The six recovered SAGs in this family show particularly high expression of sugar-binding proteins (K02025-K2027) and sugar-specific ABC transporters (TCIDs 3.A.1.1). Some SAGs also indicate transcription of peptide transporters (TCIDs 3.A.1.5), particularly *Ruminococcaceae* I18. For this SAG, up to 10% of transcripts are glycine reductase components A and B (K10670-K10672), suggestive of reductive Stickland metabolism. Separately, A16 transcribes a full suite of enzymes for catechol degradation to pyruvate (K00446, K10216, K02554, K01666). Catechol is key in insect cuticular development for the gut lining and is found in plant-derived compounds of the host’s diet (Andersen, 2010; Maini Rekdal et al., 2020). Catechol degradation demonstrates their potential for metabolic variety.

Sugar metabolism suggests a role as secondary carbohydrate degraders, primarily importing simple sugars for fermentation, as seen in human isolate *Eubacterium rectale* when grown with polysaccharide-degrading *Bacteroides thetaiotamicron* (Mahowald et al., 2009). Meanwhile, reductive Stickland metabolism harvests power by fermenting amino acids to acetate (Bouillaut et al., 2013). Together, these results suggest that in the cockroach gut, *Ruminococcaceae* are important in the metabolism of secondary substrates released from the degradation of polysaccharides and proteins by *Bacteroidota* (Figure 4).

#### 3.3.2. Christensenellales

Eight members of *Christensenellales* were found. Half of these SAGs (L17, M02, M15, N14) possess proteins identified as dockerins (K06381) that are also transcribed, albeit at relatively low levels. Two of these genomes (L17/M15) transcribe saccharide ABC transporters (TCIDs 3.A.1.1/3.A.1.2), while N14 shows high transcription of a mannose transport system (TCID 4.A.6.1.27). Similarly, transcriptional levels of glycolysis-related transcripts in *Christensenellales* SAGs are more like *Bacteroidota* SAGs than other *Firmicutes* (Figure 3), supporting their fermentation of carbohydrates and production of SCFAs (Fischbach and Sonnenburg, 2011; Flint et al., 2012).

Single cell-amplified genomes in this taxon also focus transcriptional activity on amino acid metabolism, as seen previously in members of class *Clostridia* (Barker, 1981; Fischbach and Sonnenburg, 2011). Highly transcribed ABC transporters are for peptides (TCIDs 3.A.1.5) and branched-chain amino acids (TCIDs 3.A.1.4). Six of the eight *Christensenellales* SAGs transcribe high levels of a 2-oxoacid ferredoxin oxidoreductase (K00174-6) displaying homology to 3-methyl-2-oxobutanoate dehydrogenase. This enzyme aids in catabolism of branched-chain amino acids (Bowden and Connelly, 1968). Other enzymes that degrade aromatic amino acids are also transcribed in our *Christensenellales* SAGs (K11381, K00826, K00382), supporting activity on these substrates.

*Christensenellaceae* K19—the only representative classified to the family level—displays potential for carbon fixation activity by transcribing carbon monoxide dehydrogenase (K00198) and acetyl-CoA decarbonylase/synthase (K00194) (Figure 4). Acetogenesis is a contribution often fulfilled by *Firmicutes* across a variety of hosts (Rey et al., 2010; Karekar et al., 2022). However, no formate dehydrogenase nor other enzymes in the methyl branch of the Wood-Ljungdahl pathway were found in this SAG, suggesting the organism may be coupling this metabolism to another pathway.

One potential coupling uses glycine to produce the methylene-THF substrate for acetyl-CoA synthase (Song et al., 2020). Indeed, the required enzymes for this portion—glycine dehydrogenase (K00281-3) and aminomethyltransferase (K00605)—are transcribed by K19. Using glycine in this way is reasonable, given the prevalent amino acid transporters in this SAG. Alternatively, with an estimated completeness of 21.0%, it is possible that genes for CO_2_ reduction to methylene-THF were present in the SAG but missed in sequencing. Overall, metabolic activities discussed above indicate *Christensenellales* SAGs exhibit diverse metabolic strategies, including carbohydrate fermentation, aromatic amino acid metabolism, and carbon fixation.

For *Christensenellales* A19, we see transcription of multiple phage-related proteins, including major capsid proteins and protective DNA-methyltransferases, suggesting active phage infection (Naito et al., 1995). Phage evidence is consistent with previous findings that *Firmicutes* possess more prophages than *Bacteroidota* (Jiang et al., 2019) and supports a role for phages in shaping gut microbiota (Hsu et al., 2019).

#### 3.3.3. Lachnospiraceae

Three *Lachnospiraceae* SAGs were recovered. ABC transporters are highly transcribed in this group, with specificity for sugars. Two SAGs (D06 and J05) also show potential bacterial microcompartment loci for fucose/rhamnose processing using glycyl radical-containing enzymes (Petit et al., 2013). J05 carries and expresses a complete BMC locus, and its highest transcribed ABC transporter is for fucose (TCID 3.A.1.1.47).

The fucose/rhamnose type of BMC has only been found in *Firmicutes* (Sutter et al., 2021). Fucose is a common glycan on cell wall components and mucins, including the peritrophic membrane of some insects (Liu et al., 2019). In organisms metabolizing fucose without a BMC, 1,2-propanediol is secreted and—in *Salmonella typhimurium*—recaptured and used by the organism once fucose is exhausted (Baldoma et al., 1988; Obradors et al., 1988). The BMC instead sequesters reactive intermediates produced during fucose/rhamnose fermentation and extracts the full potential from the substrate before excretion (Petit et al., 2013). Overall, these results suggest that like other members of this phylum, *Lachnospiraceae* are active in sugar metabolism and show unique metabolic strategies.

### 3.4. Desulfobacterota

In *Desulfobacterota*, SAGs fall into three groups with varying degrees of diversity: *Desulfovibrionaceae*, *Desulfobacterales*, and unclassified *Desulfobacterota*. The *Desulfovibrionaceae* appear to be represented by a moderately diverse clade associated with the *Frigididesulfovibrio* genus (Figure 2C; Waite et al., 2020). The presence of genomic diversity within *Frigididesulfovibrio* is supported by ANI results, with only two *Frigididesulfovibrio* SAGs (F20 and M16; estimated 12.2 and 28.4% complete, respectively) producing valid ANI alignments (34.75% of M16 aligned to F20 with 98.43% ANI) The remaining nine *Frigididesulfovibrio* members did not align well enough to meet inclusion criteria for ANI analysis. We do not think lack of alignment is purely due to the fragmentary nature of the SAGs, as genome completeness estimates of unaligned *Frigididesulfovibrio* are 29.3–78.2%. Of the five *Desulfobacterales* SAGs, one could not be classified further (F15), and the other four are closely related to *Desulfosarcina*. These four were found to align with one another and share 82.8–99.8% ANI over the alignment (25.8–89.2% aligned). Our unclassified *Desulfobacterota* SAGs cluster with clones from other cockroaches: this group is positioned basal to *Desulfobacterales* but diverges after *Desulfovibrionaceae*. In the 16S rRNA gene tree, this corresponds to the Rs-K70 termite group. While this group in SILVA is mostly composed of 16S rRNA gene sequences from termite hosts, our SAGs cluster in a monophyletic group with sequences from non-termite hosts including cockroaches, a scarab beetle, and angelfish (Beccati et al., 2017).

Moreso than in other phyla, transcripts with CAZyme domain GH23 (peptidoglycan lyase) are common in *Desulfobacterota*. Activity from this domain is used to remodel the cell wall for growth, division, and insertion of large structures such as flagella and secretion systems (Scheurwater et al., 2008). T6SSs are also markedly more abundant in *Desulfobacterota* members than other SAGs (Figure 3B). The majority of T6SS proteins in this phylum are similar to *Vibrionaceae*-types for antimicrobial compounds and host-interaction (TCID 3.A.23.1.1) (Dar et al., 2018). These secretion systems may be used to kill competing bacterial cells and even take up their released DNA, as is seen in *Vibrio cholerae* (Borgeaud et al., 2015). Toxins (K01406) and natural transformation genes (K02650, K02666) are indeed transcribed by our *Frigididesulfovibrio* and *Desulfosarcina* SAGs, respectively. SAGs from this phylum also display high average transcription of proteins for flagella (K02406) and two-component sensing systems (K03406, K03415), which may allow the organism to find localized sources of electron acceptors, as seen in environmental isolate, *Nitratidesulfovibrio vulgaris* Hildenborough (Ray et al., 2014).

*Desulfobacterota* in general are well known for their ability to reduce sulfate. Nearly all *Desulfobacterota* SAGs encode at least some genes required for sulfate reduction, except the genome with the fewest CDSs recovered: *Frigididesulfovibrio* F07. Many of the SAGs in this phylum transcribe high levels of sulfate reduction genes, including dissimilatory sulfite reductase (K11180/K11181), adenylylsulfate reductase (K00394/K00395), thiosulfate reductase (TCID 5.A.3.5.1), and sulfate adenylyltransferase (K00958), as well as H_2_:heterodisulfide oxidoreductases (TCID 5.B.12.1.2) thought to reduce dissimilatory sulfite reductase, DsrC (Venceslau et al., 2014).

Sources of sulfur in animal guts include sulfated amino acids from the host or its diet, as well as sulfated glycans (Carbonero et al., 2012). There is also evidence of O_2_ diffusion into the cockroach gut periphery (Bauer et al., 2015), which could potentially allow for the establishment for a complete sulfur cycle within the gut. Termite *Frigididesulfovibrio* were found to oxidize sulfide and therefore were capable of every step of the sulfur cycle (Brune et al., 1995; Dröge et al., 2005). Our *Frigididesulfovibrio* SAGs did not show evidence of sulfide oxidation genes (*soxB*, K17218), however these may have been missed in our fragmentary genomes. Given the surprisingly high levels of activity in this group and their universally high expression of sulfate reduction genes, we feel that non-dietary sources of oxidized sulfur deserve further attention.

#### 3.4.1. Frigididesulfovibrio

Eleven SAGs were classified as *Frigididesulfovibrio*. *Frigididesulfovibrio* SAGs transcribed periplasmic NiFe hydrogenases (K00437/TCID 3.D.7.2.1), lactate permease (K03303/TCID 2.A.14.2.2), and a protein mapping to lactate utilization protein B that is also annotated as a respiratory protein involved in sulfate reduction (TCID 3.D.7.1.2) (Guiral et al., 2005; Zane et al., 2010). As seen in human and environmental sulfate reducing bacteria (Zane et al., 2010; Rey et al., 2013; Tao et al., 2014), our findings indicate that our *Frigididesulfovibrio* may use both hydrogen and lactate as electron donors (Figure 4).

Hydrogen has previously been identified as a key energy intermediate in termite guts, especially in reductive acetogenesis and methanogenesis (Pester and Brune, 2007; Schauer et al., 2012). Brune and colleagues documented lactate presence and high rates of lactate turnover in termite and cockroach guts (Pester and Brune, 2007; Schauer et al., 2012). *In vitro* experiments with lactate and the human colonic isolate, *Desulfovibrio piger*, indicated use of lactate for hydrogen sulfide production (Marquet et al., 2009). Although the energy-yielding potential for lactate as an electron donor is lower than that of hydrogen (Thauer et al., 1977), *Nitratidesulfovibrio vulgaris* Hildenborough has been shown to prefer lactate over hydrogen (Tao et al., 2014). This lactate usage may be a realized niche for sulfate reduction by *Frigididesulfovibrio* in the cockroach.

A few *Frigididesulfovibrio* SAGs (G15, I05, M16) contain prevalent transcripts for glycogen metabolism (K00688). A glycogen/starch phosphorylase CAZyme domain (GT35) is moderately transcribed (Figure 3D). Glycogen use has been found in *Desulfovibrio, Nitratidesulfovibrio,* and *Megalodesulfovibrio* species from diverse environments (Stams et al., 1983) and shown as important for gut colonization in other human symbionts such as *Lactobacillus acidophilus* and *Bifidobacterium* (Goh and Klaenhammer, 2014; Milani et al., 2015). The ability to store carbon as glycogen for later use could be a competitive strategy important for these cockroach gut symbionts.

Other *Frigididesulfovibrio* were found to encode potential BMC components suggestive of taurine or choline metabolism. Two loci in *Frigididesulfovibrio* C06 (GRMguf) are proposed to break carbon-sulfur bonds as in the metabolism of taurine *via* isethionate (Peck et al., 2019). Choline processing loci are also found in *Frigididesulfovibrio* (F07, G15), potentially allowing these organisms to use choline as a sole carbon, nitrogen, and energy source. Choline trimethylamine lyase (CutC: K20038) and its activating enzyme are both transcribed in G15.

Both taurine and choline metabolism could play important roles in this system. Taurine metabolism could be a BMC strategy to produce sulfite for use as a terminal electron acceptor, as documented in the human gut microbe and sulfite reducer, *Bilophila wadsworthia*, (Xing et al., 2019; Burrichter et al., 2021). Choline metabolism has implications for the host and neighboring organisms, as this pathway produces trimethylamine (TMA) as a waste product. TMA can be converted to TMAO by host cells, which has been linked to many health risks in mammals (Li et al., 2022). TMAO has been implicated in cardiovascular disease (Wang et al., 2011). Insect monooxygenases are poorly characterized (Sehlmeyer et al., 2010), so it is unclear if *P. americana* produces the enzyme necessary for TMA conversion to TMAO (flavin-containing monooxygenase, FMO3). However, excreted methylamines have been shown to assist in cockroach aggregation (Sakuma and Fukami, 1990). Separately, TMA has also been shown to serve as a substrate for methyl-reducing methanogenesis in Archaea, including *Methanomicrococus blatticola* (Figure 4; Hippe et al., 1979; Sprenger et al., 2000). Phosphatidylcholine is found in eukaryotic cell membranes, including insects and their diet components (Shiomi et al., 2021). These specialty metabolisms enable *Frigididesulfovibrio* SAGs to use low-competition substrates for essential processes and may also support cross-feeding with methanogens.

*Desulfosarcina.* As noted above, the four *Desulfosarcina* SAGs show evidence of sulfate reducing activity as a primary energy source. Heterodisulfide reductase (Hdr)-like enzymes (K03388) are found in the top transcripts of *Desulfosarcina*, but the electron source for these enzymes is not known in this context. Distinct from *Frigididesulfovibrio,* cockroach gut *Desulfosarcina* show a potential for aromatic metabolism (Figure 3). Transcripts for benzoate degradation include glutaryl-CoA dehydrogenase (K16173), 6-oxocyclohex-1-ene-1-carbonyl-CoA hydrolase (K07539), 3-hydroxybutyryl-CoA dehydrogenase (K00074) (Härtel et al., 1993; Carmona et al., 2009).

Most studied *Desulfosarcina* species are recovered from marine environments, where they are studied for degrading aromatic compounds and hydrocarbons, including benzoate and *p*-cresol (Galushko and Rozanova, 1991; Müller et al., 2001; Savage et al., 2010). Though *Desulfosarcina* have not previously been isolated from gut environments, our analyses indicate this may be an alternative ecological niche for them, where they could utilize aromatic dietary substrates. Aromatic compounds in the gut are a by-product of aromatic amino acid fermentation, and can have neurological implications in humans (Elsden et al., 1976; Pascucci et al., 2020).

As a whole, *Desulfobacteraceae* (including *Desulfosarcina*) is one of the most active families in this dataset (as defined by RNA:DNA ratio; Figure 1C) and makes up the largest proportion of metatranscriptome reads of any clade (Figure 1B). This is surprising based on this family’s abundances in 16S rRNA gene datasets from cockroaches and humans: approximately 0–3.4% (Pérez-Cobas et al., 2015; Tinker and Ottesen, 2016; Kiernan et al., 2021). It suggests that *Desulfosarcina* are highly active and may contribute more extensively to the function of the cockroach gut community than previously expected.

#### 3.4.2. Unclassified *Desulfobacterota*

The two SAGs in this group dedicate smaller proportions of top transcripts to sulfate reduction than do *Frigididesulfovibrio* and *Desulfosarcina*. Rs-K70 contains references identified as potentially responsible for reductive acetogenesis in termite and cockroach guts (Rosenthal et al., 2013; Ikeda-Ohtsubo et al., 2016). References in the 16S and genome tree include a homoacetogenic bacterial endosymbiont localized to a *Trichonympha* flagellate from termite guts (Ikeda-Ohtsubo et al., 2016), indicating that our unclassified *Desulfobacterota* SAGs may fulfill a similar function. As such, transcripts for carbon monoxide dehydrogenase (K00198), formate dehydrogenase (K22015), formate-tetrahydrofolate ligase (K01938), 5-methyltetrahydrofolate corrinoid/iron sulfur protein methyltransferase (K15023), methenyltetrahydrofolate cyclohydrolase (K01500), and acetyl-CoA synthase (K14138) are expressed in this group, supporting active use of the Wood-Ljungdahl pathway.

*Desulfobacterota* M10 also has a highly transcribed BMC locus for ethanolamine metabolism, including transcription of its signature enzyme, ethanolamine ammonia-lyase (K03735/K03736). A similar locus in *E. coli* is thought to enable that bacterium to utilize ethanolamine as a nitrogen source for a competitive advantage (Bertin et al., 2011). Together, these results suggest that these *Desulfobacterota* complement sulfur compound reduction with metabolism of alternative carbon and nitrogen substrates. Differences in reference genome isolation source and characterized metabolic activities of references in this phylum suggest diverse evolutionary pathways and potential for specialization in sulfate-reducing bacteria of the cockroach gut.

### 3.5. Methanogenic Archaea

This dataset includes two discrete groups of methanogenic Archaea: *Methanobacteriota* (*Methanobrevibacter*) and *Halobacteriota* (*Methanimicrococcus*). The two *Methanobrevibacter* SAGs are nearly clonal, sharing an ANI >99.9% over an alignment that covers 70% of their genomes (Supplementary Table S1-7). *Methanobrevibacter* I08 is estimated as this dataset’s most-complete SAG at 86%. Though *Methanobrevibacter* species are well-known human gut archaea (Miller et al., 1982; Saengkerdsub and Ricke, 2014; Lloyd-Price et al., 2016), our SAGs’ 16S rRNA gene sequences indicate closer relation to an isolate from an anaerobic digester than to human-associated *M. smithii* (Savant et al., 2002). The *Methanimicroccocus* SAG (J09) is similar to *Methanimicrococcus blatticola*, also isolated from *P. americana* (Sprenger et al., 2000), but other references for this genus are non-host-associated, illustrating a gap in understanding of these organisms in a gut environment. Though *Methanimicrococcus* is not a prominent member of the human gut microbiome, this SAG is more prevalent than *Methanobrevibacter* in cockroach-derived 16S, metagenome, and metatranscriptome datasets and displays the highest average relative activity of any one SAG (Figure 1). Representation of two methanogenic taxa offers another opportunity to look at differences between taxa from the same functional group.

As expected, given the specialization of this group, all archaeal SAGs display many methanogenesis transcripts, and the H_2_: heterodisulfide reductase (Hdr) is present in all three SAGs. Very few CAZymes are found in our methanogenic archaea, as seen before (Samuel et al., 2007). The most prevalent domain transcribed is GT2, and this is the only CAZyme domain found in *Methanimicrococcus*. In methanogens, this domain is thought to aid in host mimicry and the production of N-acetylneuraminic acid (Neu5Ac), the predominant type of sialic acid found in human mucus/epithelial surface glycans (Samuel et al., 2007). Though these clades of methanogens complete similar functions, their transcriptional activities indicate distinct, functional specialization.

#### 3.5.1. Methanobrevibacter

In addition to methanogenesis-related transcripts, both *Methanobrevibacter* SAGs transcribe hypothetical proteins that show homology to immunoglobulin-like proteins in other *Methanobrevibacter* species. Immunoglobulin-like proteins may be involved in adhesion (Ng et al., 2016). Our SAGs also transcribe GH109, a domain key in glycan degradation *via* alpha-*N*-acetylgalactosaminidase and removal of *O*-glycans (Liu et al., 2007).

These results are suggestive of adhesion and host-association. Studies have identified *Methanobrevibacter* as potentially associated with the hindgut wall in cockroaches and termites (Hackstein and Stumm, 1994; Brune, 2019). A related species by 16S rRNA phylogeny—*Methanobrevibacter* sp. *LHD12*—has been shown to be associated with flagellates in the termite gut (Tokura et al., 2000). Finally, a study comparing the human isolate *M. smithii* to non-gut methanogens found similar results that human isolate *M. smithii* is well equipped to mimic host glycans (using GT2) and adhere to host structures, such as mucosal glycosaminoglycans (Samuel et al., 2007).

*Methanobrevibacter* SAGs also show transcripts for formate dehydrogenase (K22516), potentially indicating the use of formate—in addition to hydrogen—as an electron donor for methanogenesis as described for rumen *Methanobrevibacter* species in Rea et al. (2007).

#### 3.5.2. Methanimicrococcus

The *Methanimicrococcus* SAG recovered shares 83% ANI to the relatively well-characterized cockroach isolate *Methanimicrococcus blatticola* (Sprenger et al., 2000; Thomas et al., 2021). Our transcriptional data show high expression of methanophenazine hydrogenase (K14070), methylamine transporters (TCIDs 2.A.3.5.2, 2.A.3.6.2), and methyl transferases (monomethylamine/dimethylamine/methanol) (K16176, K16177, K16178, K04480, K14082), enabling *Methanimicrocccus* to use these compounds rather than CO_2_ for methanogenesis, as described in Kurth et al. (2020).

*Methanimicrococcus* differentiates itself from *Methanobrevibacter* by using organic carbon sources for methanogenesis and an inorganic electron donor, while *Methanobrevibacter* can use inorganic carbon and an organic electron donor. *Methanimicrococcus blatticola* lacks genes to utilize the H_4_MPT methyl branch of the pathway to reduce CO_2_ (Sprenger et al., 2005). Instead, it can utilize methanol and methylamines. This specialization likely prevents competition over shared substrates, and methanol and methylamine usage provides a cross-feeding opportunity with polysaccharide and choline degraders (Figure 4).

## 4. Conclusion

We used single-cell genome and metatranscriptome sequencing to discover phylogeny, genomic content, and transcriptional activities of individual uncultured organisms in the cockroach hindgut environment. With these data, we develop a model identifying potential roles for these organisms in cockroach hindgut metabolism (Figure 4). Our data suggest that the taxonomic partitioning of metabolic functions in the cockroach gut is broadly consistent with that observed in mammalian gut environments, particularly at the phylum level. However, we have also generated novel insights into metabolic specialization of different lineages at the family and genus level, as well as the functional roles of insect-specific lineages in *Bacteroidota* and *Desulfobacterota*.

Our *Bacteroidota* data are largely consistent with their canonical roles as carbohydrate degraders with transcriptional patterns for different genera showing divergent specialties and metabolic priorities. An exception is a group of unclassified *Bacteroidales* that was found to exhibit high transcriptional activities in genes involved in cell–cell interaction and gliding motility. This evolutionarily divergent clade is well-represented in molecular surveys of cockroaches and termites, but this is the first description of its functional capabilities.

SAGs assigned to *Firmicutes* display extensive diversity of phylogenetic history, transcriptional functions, and metabolic potential. No two *Firmicutes* SAGs were placed in the same genus, and many SAGs cannot be classified beyond family level. This group showed high expression of DNA transfer, transposon, and phage proteins. The transcription of mobile genetic elements suggests movement of genes in the population, potentially explaining their metabolic and genomic diversity. Given this diversity and low redundancy, it is difficult to fully characterize the functional capabilities of cockroach gut *Firmicutes*. However, our single-cell analysis with transcript sequences offers a rare look into *Firmicutes* activity *in vivo,* including autotrophy and secondary degradation of sugars and peptides released from polysaccharides.

An unexpected finding of our work was the high abundance and activity levels of *Desulfobacterota*. While common in 16S rRNA gene datasets, they are often at moderately low levels (0–3.2%). However, their abundance in our transcriptional data suggests that this population may be highly active. Electron donors and acceptors for sulfate reduction are key products and substrates from other community members including glycan degraders, methanogens, hydrogenotrophs, and carbohydrate fermenters. We also identified *Desulfobacterota* closely related to the Rs-K70 insect-associated clade carrying ethanolamine and choline BMCs, which have implications for the host and peer microbiota. Our findings that these bacteria are highly active and more abundant than previously predicted have important implications for cockroach gut metabolism.

Methanogenesis is a shared key function for our archaeal SAGs, however distinct clades show unique strategies. *Methanimicrococcus* indicates use of methylamines and methanol rather than CO_2_, catabolizing compounds that are precursors to toxins (TMA/TMAO). *Methanobrevibacter* SAGs meanwhile express components to use a formate electron donor for methanogenesis. Like their mammalian relatives, cockroach methanogens use CAZymes for producing host-epithelial glycans and—for *Methanobrevibacter*—metabolizing *O*-glycans to take advantage of the host ecosystem while carrying out methanogenesis.

Phylogenetic analyses indicate that many cockroach gut symbionts represent new species and genera that bridge environmental and host-associated clades. Despite distinct evolutionary histories of cockroach gut SAGs and human gut isolates, the microbiota from these hosts indicate shared metabolic strategies in their host-association. Furthermore, by capturing multiple SAGs of the same genus we define transcriptional functions for the group and illuminate niches within functional groups (Figure 4). This work opens avenues for microbiome hypothesis development, directed experiments, and novel species isolation efforts.

## Data availability statement

The datasets presented in this study can be found in online repositories. The names of the repository/repositories and accession number(s) can be found in the article/Supplementary material.

## Author contributions

HD prepared the sample for single-cell genome sequencing, prepared the metagenomic libraries from DNA samples, carried out bioinformatic analyses, interpreted the data, and drafted the manuscript. KT cared for and collected cockroach samples, dissected insect guts, extracted RNA and DNA for metatranscriptome and metagenome sequencing, respectively, prepared metatranscriptome libraries for sequencing, and assisted in manuscript editing. EO contributed as principal supervisor and project coordinator and participated in all aspects of project design, data interpretation, and critical manuscript revisions. All authors have read and approved this manuscript.

## Funding

This research was supported by the National Institute of General Medical Sciences of the National Institutes of Health (NIH) under award number R35GM133789.

## Conflict of interest

The authors declare that the research was conducted in the absence of any commercial or financial relationships that could be construed as a potential conflict of interest.

## Publisher’s note

All claims expressed in this article are solely those of the authors and do not necessarily represent those of their affiliated organizations, or those of the publisher, the editors and the reviewers. Any product that may be evaluated in this article, or claim that may be made by its manufacturer, is not guaranteed or endorsed by the publisher.

## Footnotes

^1^ https://github.com/fhcrc/taxtastic

## References

[ref1] Amaretti A. Gozzoli C. Simone M. Raimondi S. Righini L. Pérez-Brocal V.. (2019). Profiling of protein degraders in cultures of human gut microbiota. Front. Microbiol. 10:2614. doi: 10.3389/fmicb.2019.02614 31803157 PMC6874058

[ref2] Andersen S. O. (2010). Insect cuticular sclerotization: a review. Insect Biochem. Mol. Biol. 40, 166–178. doi: 10.1016/j.ibmb.2009.10.007 19932179

[ref3] Aramaki T. Blanc-Mathieu R. Endo H. Ohkubo K. Kanehisa M. Goto S.. (2020). KofamKOALA: KEGG ortholog assignment based on profile HMM and adaptive score threshold. Bioinformatics 36, 2251–2252. doi: 10.1093/bioinformatics/btz859 31742321 PMC7141845

[ref4] Baldoma L. Badia J. Obradors N. Aguilar J. (1988). Aerobic excretion of 1,2-propanediol by *Salmonella typhimurium*. J. Bacteriol. 170, 2884–2885. doi: 10.1128/jb.170.6.2884-2885.1988 3286623 PMC211220

[ref5] Barcenilla A. Pryde S. E. Martin J. C. Duncan S. H. Stewart C. S. Henderson C.. (2000). Phylogenetic relationships of butyrate-producing bacteria from the human gut. Appl. Environ. Microbiol. 66, 1654–1661. doi: 10.1128/AEM.66.4.1654-1661.2000 10742256 PMC92037

[ref6] Barker H. A. (1981). Amino acid degradation by anaerobic bacteria. Annu. Rev. Biochem. 50, 23–40. doi: 10.1146/annurev.bi.50.070181.000323 6791576

[ref7] Bauer E. Lampert N. Mikaelyan A. Köhler T. Maekawa K. Brune A. (2015). Physicochemical conditions, metabolites and community structure of the bacterial microbiota in the gut of wood-feeding cockroaches (Blaberidae: Panesthiinae). FEMS Microbiol. Ecol. 91, 1–14. doi: 10.1093/femsec/fiu028, PMID: 25764554

[ref8] Beccati A. Gerken J. Quast C. Yilmaz P. Glöckner F. O. (2017). SILVA tree viewer: interactive web browsing of the SILVA phylogenetic guide trees. BMC Bioinformatics 18:433. doi: 10.1186/s12859-017-1841-3 28964270 PMC5622420

[ref9] Bengtsson J. Eriksson K. M. Hartmann M. Wang Z. Shenoy B. D. Grelet G.-A.. (2011). Metaxa: a software tool for automated detection and discrimination among ribosomal small subunit (12S/16S/18S) sequences of archaea, bacteria, eukaryotes, mitochondria, and chloroplasts in metagenomes and environmental sequencing datasets. Antonie Van Leeuwenhoek 100, 471–475. doi: 10.1007/s10482-011-9598-6, PMID: 21674231

[ref10] Bertin Y. Girardeau J. P. Chaucheyras-Durand F. Lyan B. Pujos-Guillot E. Harel J.. (2011). Enterohaemorrhagic *Escherichia coli* gains a competitive advantage by using ethanolamine as a nitrogen source in the bovine intestinal content. Environ. Microbiol. 13, 365–377. doi: 10.1111/j.1462-2920.2010.02334.x 20849446

[ref11] Böck D. Medeiros J. M. Tsao H.-F. Penz T. Weiss G. L. Aistleitner K.. (2017). In situ architecture, function, and evolution of a contractile injection system. Science (New York, N.Y.) 357, 713–717. doi: 10.1126/science.aan7904 28818949 PMC6485382

[ref12] Borgeaud S. Metzger L. C. Scrignari T. Blokesch M. (2015). The type VI secretion system of *Vibrio cholerae* fosters horizontal gene transfer. Science 347, 63–67. doi: 10.1126/science.1260064 25554784

[ref13] Bouillaut L. Self W. T. Sonenshein A. L. (2013). Proline-dependent regulation of *Clostridium difficile* Stickland metabolism. J. Bacteriol. 195, 844–854. doi: 10.1128/JB.01492-12 23222730 PMC3562115

[ref14] Bowden J. A. Connelly J. L. (1968). Branched chain α-keto acid metabolism: II. Evidence for the common identity of α-ketoisocaproic acid and α-keto-β-methyl-valeric acid dehydrogenases. J. Biol. Chem. 243, 3526–3531. doi: 10.1016/S0021-9258(18)93339-9 5656388

[ref15] Breton C. Šnajdrová L. Jeanneau C. Koča J. Imberty A. (2005). Structures and mechanisms of glycosyltransferases. Glycobiology 16, 29R–37R. doi: 10.1093/glycob/cwj016 16037492

[ref16] Brune A. (2019). Methanogenesis in the digestive tracts of insects and other arthropods Springer International Publishing, 229–260.

[ref17] Brune A. Emerson D. Breznak J. A. (1995). The termite gut microflora as an oxygen sink: microelectrode determination of oxygen and pH gradients in guts of lower and higher termites. Appl. Environ. Microbiol. 61, 2681–2687. doi: 10.1128/aem.61.7.2681-2687.1995 16535076 PMC1388494

[ref18] Buchfink B. Xie C. Huson D. H. (2015). Fast and sensitive protein alignment using DIAMOND. Nat. Methods 12, 59–60. doi: 10.1038/nmeth.3176 25402007

[ref19] Burrichter A. G. Dörr S. Bergmann P. Haiß S. Keller A. Fournier C.. (2021). Bacterial microcompartments for isethionate desulfonation in the taurine-degrading human-gut bacterium *Bilophila wadsworthia*. BMC Microbiol. 21:340. doi: 10.1186/s12866-021-02386-w, PMID: 34903181 PMC8667426

[ref20] Callahan B. J. Mcmurdie P. J. Rosen M. J. Han A. W. Johnson A. J. A. Holmes S. P. (2016). DADA2: high-resolution sample inference from Illumina amplicon data. Nat. Methods 13, 581–583. doi: 10.1038/nmeth.3869 27214047 PMC4927377

[ref21] Capella-Gutiérrez S. Silla-Martínez J. M. Gabaldón T. (2009). TrimAl: a tool for automated alignment trimming in large-scale phylogenetic analyses. Bioinformatics 25, 1972–1973. doi: 10.1093/bioinformatics/btp348 19505945 PMC2712344

[ref22] Carbonero F. Benefiel A. Alizadeh-Ghamsari A. Gaskins H. R. (2012). Microbial pathways in colonic sulfur metabolism and links with health and disease. Front. Physiol. 3:448. doi: 10.3389/fphys.2012.00448 23226130 PMC3508456

[ref23] Carmona M. Zamarro M. A. T. Blázquez B. Durante-Rodríguez G. JuáRez J. F. Valderrama J. A. S.. (2009). Anaerobic catabolism of aromatic compounds: a genetic and genomic view. Microbiol. Mol. Biol. Rev. 73, 71–133. doi: 10.1128/mmbr.00021-08 19258534 PMC2650882

[ref24] Chow V. Nong G. Preston J. F. (2007). Structure, function, and regulation of the aldouronate utilization gene cluster from *Paenibacillus* sp. strain JDR-2. J. Bacteriol. 189, 8863–8870. doi: 10.1128/jb.01141-07 17921311 PMC2168633

[ref25] Ciufo S. Kannan S. Sharma S. Badretdin A. Clark K. Turner S.. (2018). Using average nucleotide identity to improve taxonomic assignments in prokaryotic genomes at the NCBI. Int. J. Syst. Evol. Microbiol. 68, 2386–2392. doi: 10.1099/ijsem.0.002809 29792589 PMC6978984

[ref26] Clark K. Karsch-Mizrachi I. Lipman D. J. Ostell J. Sayers E. W. (2016). GenBank. Nucleic Acids Res. 44, D67–D72. doi: 10.1093/nar/gkv1276 26590407 PMC4702903

[ref27] Cockburn D. W. Koropatkin N. M. (2016). Polysaccharide degradation by the intestinal microbiota and its influence on human health and disease. J. Mol. Biol. 428, 3230–3252. doi: 10.1016/j.jmb.2016.06.021 27393306

[ref28] Dar Y. Salomon D. Bosis E. (2018). The antibacterial and anti-eukaryotic type VI secretion system MIX-effector repertoire in Vibrionaceae. Mar. Drugs 16:433. doi: 10.3390/md16110433 30400344 PMC6267618

[ref29] Dietrich C. Köhler T. Brune A. (2014). The cockroach origin of the termite gut microbiota: patterns in bacterial community structure reflect major evolutionary events. Appl. Environ. Microbiol. 80, 2261–2269. doi: 10.1128/aem.04206-13 24487532 PMC3993134

[ref30] Dröge S. Limper U. Emtiazi F. Schönig I. Pavlus N. Drzyzga O.. (2005). In vitro and in vivo sulfate reduction in the gut contents of the termite *Mastotermes darwiniensis* and the rose-chafer *Pachnoda marginata*. J. Gen. Appl. Microbiol. 51, 57–64. doi: 10.2323/jgam.51.57 15942866

[ref31] Dukes H. E. Dyer J. E. Ottesen E. A. (2021). Establishment and maintenance of gnotobiotic American cockroaches (*Periplaneta americana*). JoVE e61316:e61316. doi: 10.3791/61316, PMID: 34125088 PMC9009305

[ref32] Eckburg P. B. Bik E. M. Bernstein C. N. Purdom E. Dethlefsen L. Sargent M.. (2005). Diversity of the human intestinal microbial flora. Science 308, 1635–1638. doi: 10.1126/science.1110591 15831718 PMC1395357

[ref33] Eddy S. R. (2011). Accelerated profile HMM searches. PLoS Comput. Biol. 7:e1002195. doi: 10.1371/journal.pcbi.1002195 22039361 PMC3197634

[ref34] Eddy S.R. (2020). HMMer 3.3.2 (Nov 2020). Available at: http://hmmer.org/

[ref35] Elsden S. R. Hilton M. G. Waller J. M. (1976). The end products of the metabolism of aromatic amino acids by Clostridia. Arch. Microbiol. 107, 283–288. doi: 10.1007/BF00425340 1275638

[ref36] Eren A. M. Kiefl E. Shaiber A. Veseli I. Miller S. E. Schechter M. S.. (2021). Community-led, integrated, reproducible multi-omics with Anvi’o. Nat. Microbiol. 6, 3–6. doi: 10.1038/s41564-020-00834-3 33349678 PMC8116326

[ref37] Falony G. Vlachou A. Verbrugghe K. Vuyst L. D. (2006). Cross-feeding between *Bifidobacterium longum* BB536 and acetate-converting, butyrate-producing colon bacteria during growth on oligofructose. Appl. Environ. Microbiol. 72, 7835–7841. doi: 10.1128/aem.01296-06 17056678 PMC1694233

[ref38] Fernando D. Kumar A. (2013). Resistance-nodulation-division multidrug efflux pumps in gram-negative bacteria: role in virulence. Antibiotics 2, 163–181. doi: 10.3390/antibiotics2010163, PMID: 27029297 PMC4790303

[ref39] Fischbach M. A. Sonnenburg J. L. (2011). Eating for two: how metabolism establishes interspecies interactions in the gut. Cell Host Microbe 10, 336–347. doi: 10.1016/j.chom.2011.10.002 22018234 PMC3225337

[ref40] Flint H. J. Scott K. P. Duncan S. H. Louis P. Forano E. (2012). Microbial degradation of complex carbohydrates in the gut. Gut Microbes 3, 289–306. doi: 10.4161/gmic.19897, PMID: 22572875 PMC3463488

[ref41] Galushko A. Rozanova E. (1991). *Desulfobacterium cetonicum* sp. nov., a sulfate-reducing bacterium which oxidizes fatty acids and ketones. Microbiology 60, 742–746.

[ref42] Garcia J.-L. Patel B. K. C. Ollivier B. (2000). Taxonomic, phylogenetic, and ecological diversity of methanogenic archaea. Anaerobe 6, 205–226. doi: 10.1006/anae.2000.0345 16887666

[ref43] Glöckner F. O. Yilmaz P. Quast C. Gerken J. Beccati A. Ciuprina A. (2017). 25 years of serving the community with ribosomal RNA gene reference databases and tools. J. Biotechnol. 261, 169–176. doi: 10.1016/j.jbiotec.2017.06.1198 28648396

[ref44] Goh Y. J. Klaenhammer T. R. (2014). Insights into glycogen metabolism in *Lactobacillus acidophilus*: impact on carbohydrate metabolism, stress tolerance and gut retention. Microb. Cell Factories 13:94. doi: 10.1186/s12934-014-0094-3 PMC4243779 25410006

[ref45] Guiral M. Tron P. Aubert C. Gloter A. Iobbi-Nivol C. Giudici-Orticoni M.-T. (2005). A membrane-bound multienzyme, hydrogen-oxidizing, and sulfur-reducing complex from the hyperthermophilic bacterium *Aquifex aeolicus*. J. Biol. Chem. 280, 42004–42015. doi: 10.1074/jbc.m508034200 16236714

[ref46] Guo P. Zhang K. Ma X. He P. (2020). Clostridium species as probiotics: potentials and challenges. J. Anim. Sci. Biotechnol. 11:24. doi: 10.1186/s40104-019-0402-1, PMID: 32099648 PMC7031906

[ref47] Hackstein J. H. Stumm C. K. (1994). Methane production in terrestrial arthropods. Proc. Natl. Acad. Sci. 91, 5441–5445. doi: 10.1073/pnas.91.12.5441, PMID: 8202505 PMC44011

[ref48] Härtel U. Eckel E. Koch J. Fuchs G. Linder D. Buckel W. (1993). Purification of glutaryl-CoA dehydrogenase from *Pseudomonas* sp., an enzyme involved in the anaerobic degradation of benzoate. Arch. Microbiol. 159, 174–181. doi: 10.1007/bf00250279, PMID: 8439237

[ref49] Harvey A. J. Hrmova M. De Gori R. Varghese J. N. Fincher G. B. (2000). Comparative modeling of the three-dimensional structures of family 3 glycoside hydrolases. Proteins: structure. Funct. Bioinformatics 41, 257–269. doi: 10.1002/1097-0134(20001101)41:2<257::AID-PROT100>3.0.CO;2-C 10966578

[ref50] Hedblom G. A. Reiland H. A. Sylte M. J. Johnson T. J. Baumler D. J. (2018). Segmented filamentous bacteria – metabolism meets immunity. Front. Microbiol. 9:1991. doi: 10.3389/fmicb.2018.01991 30197636 PMC6117376

[ref51] Hippe H. Caspari D. Fiebig K. Gottschalk G. (1979). Utilization of trimethylamine and other N-methyl compounds for growth and methane formation by *Methanosarcina barkeri*. Proc. Natl. Acad. Sci. 76, 494–498. doi: 10.1073/pnas.76.1.494 284366 PMC382968

[ref52] Hongoh Y. Sato T. Noda S. Ui S. Kudo T. Ohkuma M. (2007). Candidatus Symbiothrix dinenymphae: bristle-like Bacteroidales ectosymbionts of termite gut protists. Environ. Microbiol. 9, 2631–2635. doi: 10.1111/j.1462-2920.2007.01365.x 17803785

[ref53] Hongoh Y. Sharma V. K. Prakash T. Noda S. Toh H. Taylor T. D.. (2008). Genome of an endosymbiont coupling N_2_ fixation to cellulolysis within protist cells in termite gut. Science 322, 1108–1109. doi: 10.1126/science.1165578 19008447

[ref54] Hsu B. B. Gibson T. E. Yeliseyev V. Liu Q. Lyon L. Bry L.. (2019). Dynamic modulation of the gut microbiota and metabolome by bacteriophages in a mouse model. Cell Host Microbe 25, 803–814.e805. doi: 10.1016/j.chom.2019.05.001 31175044 PMC6579560

[ref55] Ikeda-Ohtsubo W. Strassert J. F. H. Köhler T. Mikaelyan A. Gregor I. Mchardy A. C.. (2016). ‘Candidatus Adiutrix intracellularis’, an endosymbiont of termite gut flagellates, is the first representative of a deep-branching clade of Deltaproteobacteria and a putative homoacetogen. Environ. Microbiol. 18, 2548–2564. doi: 10.1111/1462-2920.13234 26914459

[ref56] Inoue J.-I. Oshima K. Suda W. Sakamoto M. Iino T. Noda S.. (2015). Distribution and evolution of nitrogen fixation genes in the phylum Bacteroidetes. Microbes Environ. 30, 44–50. doi: 10.1264/jsme2.me14142 25736980 PMC4356463

[ref57] Jahnes B. C. Herrmann M. Sabree Z. L. (2019). Conspecific coprophagy stimulates normal development in a germ-free model invertebrate. PeerJ 7:e6914. doi: 10.7717/peerj.6914 31139506 PMC6521811

[ref58] Jahnes B. C. Sabree Z. L. (2020). Nutritional symbiosis and ecology of host-gut microbe systems in the Blattodea. Curr. Opin. Insect Sci. 39, 35–41. doi: 10.1016/j.cois.2020.01.001 32109859

[ref59] Jain C. Rodriguez R. L. M. Phillippy A. M. Konstantinidis K. T. Aluru S. (2018). High throughput ANI analysis of 90K prokaryotic genomes reveals clear species boundaries. Nat. Commun. 9:5114. doi: 10.1038/s41467-018-07641-9 30504855 PMC6269478

[ref60] Jiang X. Hall A. B. Xavier R. J. Alm E. J. (2019). Comprehensive analysis of chromosomal mobile genetic elements in the gut microbiome reveals phylum-level niche-adaptive gene pools. PLoS One 14:e0223680. doi: 10.1371/journal.pone.0223680 31830054 PMC6907783

[ref61] Kanehisa M. (2000). KEGG: Kyoto encyclopedia of genes and genomes. Nucleic Acids Res. 28, 27–30. doi: 10.1093/nar/28.1.27 10592173 PMC102409

[ref62] Kanehisa M. (2019). Toward understanding the origin and evolution of cellular organisms. Protein Sci. 28, 1947–1951. doi: 10.1002/pro.3715 31441146 PMC6798127

[ref63] Kanehisa M. Furumichi M. Sato Y. Ishiguro-Watanabe M. Tanabe M. (2021). KEGG: integrating viruses and cellular organisms. Nucleic Acids Res. 49, D545–D551. doi: 10.1093/nar/gkaa970 33125081 PMC7779016

[ref64] Karekar S. Stefanini R. Ahring B. (2022). Homo-acetogens: their metabolism and competitive relationship with hydrogenotrophic methanogens. Microorganisms 10:397. doi: 10.3390/microorganisms10020397 35208852 PMC8875654

[ref65] Kiernan M. G. Dunne S. S. Dunne C. P. (2021). Emergence of the human gut microbiota as an influencer in health and disease Springer International Publishing, 43–51.

[ref66] Kurth J. M. Op Den Camp H. J. M. Welte C. U. (2020). Several ways one goal—methanogenesis from unconventional substrates. Appl. Microbiol. Biotechnol. 104, 6839–6854. doi: 10.1007/s00253-020-10724-7 32542472 PMC7374477

[ref67] Kvist T. Ahring B. K. Lasken R. S. Westermann P. (2007). Specific single-cell isolation and genomic amplification of uncultured microorganisms. Appl. Microbiol. Biotechnol. 74, 926–935. doi: 10.1007/s00253-006-0725-7, PMID: 17109170

[ref68] Lampert N. Mikaelyan A. Brune A. (2019). Diet is not the primary driver of bacterial community structure in the gut of litter-feeding cockroaches. BMC Microbiol. 19:238. doi: 10.1186/s12866-019-1601-9 31666028 PMC6864750

[ref69] Latasa C. Roux A. Toledo-Arana A. Ghigo J.-M. Gamazo C. Penadés J. R.. (2005). BapA, a large secreted protein required for biofilm formation and host colonization of *Salmonella enterica* serovar Enteritidis. Mol. Microbiol. 58, 1322–1339. doi: 10.1111/j.1365-2958.2005.04907.x 16313619

[ref70] León A. V.-P. D. Jahnes B. C. Otero-Bravo A. Sabree Z. L. (2021). Microbiota perturbation or elimination can inhibit normal development and elicit a starvation-like response in an omnivorous model invertebrate. mSystems 6, e00802–e00821. doi: 10.1128/mSystems.00802-21 34427529 PMC8407121

[ref71] Li D. Lu Y. Yuan S. Cai X. He Y. Chen J.. (2022). Gut microbiota–derived metabolite trimethylamine-N-oxide and multiple health outcomes: an umbrella review and updated meta-analysis. Am. J. Clin. Nutr. 116, 230–243. doi: 10.1093/ajcn/nqac074 35348578 PMC9257469

[ref72] Li S. Zhu S. Jia Q. Yuan D. Ren C. Li K.. (2018). The genomic and functional landscapes of developmental plasticity in the American cockroach. Nat. Commun. 9:1008. doi: 10.1038/s41467-018-03281-1 29559629 PMC5861062

[ref73] Liu D. De Schutter K. Smargiasso N. De Pauw E. Van Damme E. J. M. Smagghe G. (2019). The N-glycan profile of the peritrophic membrane in the Colorado potato beetle larva (*Leptinotarsa decemlineata*). J. Insect Physiol. 115, 27–32. doi: 10.1016/j.jinsphys.2019.03.009 30935980

[ref74] Liu Q. P. Sulzenbacher G. Yuan H. Bennett E. P. Pietz G. Saunders K.. (2007). Bacterial glycosidases for the production of universal red blood cells. Nat. Biotechnol. 25, 454–464. doi: 10.1038/nbt1298 17401360

[ref75] Lloyd-Price J. Abu-Ali G. Huttenhower C. (2016). The healthy human microbiome. Genome Med. 8:51. doi: 10.1186/s13073-016-0307-y 27122046 PMC4848870

[ref76] Lozupone C. A. Stombaugh J. I. Gordon J. I. Jansson J. K. Knight R. (2012). Diversity, stability and resilience of the human gut microbiota. Nature 489, 220–230. doi: 10.1038/nature11550, PMID: 22972295 PMC3577372

[ref77] Macy J. M. Ljungdahl L. G. Gottschalk G. (1978). Pathway of succinate and propionate formation in *Bacteroides fragilis*. J. Bacteriol. 134, 84–91. doi: 10.1128/jb.134.1.84-91.1978 148460 PMC222221

[ref78] Mahowald M. A. Rey F. E. Seedorf H. Turnbaugh P. J. Fulton R. S. Wollam A.. (2009). Characterizing a model human gut microbiota composed of members of its two dominant bacterial phyla. Proc. Natl. Acad. Sci. 106, 5859–5864. doi: 10.1073/pnas.0901529106 19321416 PMC2660063

[ref79] Maini Rekdal V. Nol Bernadino P. Luescher M. U. Kiamehr S. Le C. Bisanz J. E.. (2020). A widely distributed metalloenzyme class enables gut microbial metabolism of host-and diet-derived catechols. eLife:9. doi: 10.7554/elife.50845 PMC7028382 32067637

[ref80] Malmstrom R. R. Rodrigue S. Huang K. H. Kelly L. Kern S. E. Thompson A.. (2013). Ecology of uncultured Prochlorococcus clades revealed through single-cell genomics and biogeographic analysis. ISME J. 7, 184–198. doi: 10.1038/ismej.2012.89 22895163 PMC3526172

[ref81] Marquet P. Duncan S. H. Chassard C. Bernalier-Donadille A. Flint H. J. (2009). Lactate has the potential to promote hydrogen sulphide formation in the human colon. FEMS Microbiol. Lett. 299, 128–134. doi: 10.1111/j.1574-6968.2009.01750.x 19732152

[ref82] Martens E. C. Chiang H. C. Gordon J. I. (2008). Mucosal glycan foraging enhances fitness and transmission of a saccharolytic human gut bacterial symbiont. Cell Host Microbe 4, 447–457. doi: 10.1016/j.chom.2008.09.007 18996345 PMC2605320

[ref83] Matsen F. A. Kodner R. B. Armbrust E. V. (2010). Pplacer: linear time maximum-likelihood and Bayesian phylogenetic placement of sequences onto a fixed reference tree. BMC Bioinform. 11:538. doi: 10.1186/1471-2105-11-538 PMC3098090 21034504

[ref84] Mcbride M. J. (2019). Bacteroidetes gliding motility and the type IX secretion system. Microbiology. Spectrum 7:7.1.15. doi: 10.1128/microbiolspec.PSIB-0002-2018 PMC11588200 30767845

[ref85] Meyer F. Fritz A. Deng Z.-L. Koslicki D. Lesker T. R. Gurevich A.. (2022). Critical assessment of metagenome interpretation: the second round of challenges. Nat. Methods 19, 429–440. doi: 10.1038/s41592-022-01431-4, PMID: 35396482 PMC9007738

[ref86] Mikaelyan A. Köhler T. Lampert N. Rohland J. Boga H. Meuser K.. (2015). Classifying the bacterial gut microbiota of termites and cockroaches: a curated phylogenetic reference database (DictDb). Syst. Appl. Microbiol. 38, 472–482. doi: 10.1016/j.syapm.2015.07.004 26283320

[ref87] Mikaelyan A. Thompson C. L. Hofer M. J. Brune A. (2016). Deterministic assembly of complex bacterial communities in guts of germ-free cockroaches. Appl. Environ. Microbiol. 82, 1256–1263. doi: 10.1128/AEM.03700-15 26655763 PMC4751828

[ref88] Milani C. Lugli G. A. Duranti S. Turroni F. Mancabelli L. Ferrario C.. (2015). Bifidobacteria exhibit social behavior through carbohydrate resource sharing in the gut. Sci. Rep. 5:15782. doi: 10.1038/srep15782 26506949 PMC4623478

[ref89] Miller T. L. Wolin M. J. De Macario E. C. Macario A. J. (1982). Isolation of *Methanobrevibacter smithii* from human feces. Appl. Environ. Microbiol. 43, 227–232. doi: 10.1128/aem.43.1.227-232.1982 6798932 PMC241804

[ref90] Müller J. A. Galushko A. S. Kappler A. Schink B. (2001). Initiation of anaerobic degradation of p-cresol by formation of 4-hydroxybenzylsuccinate in *Desulfobacterium cetonicum*. J. Bacteriol. 183, 752–757. doi: 10.1128/JB.183.2.752-757.2001, PMID: 11133971 PMC94933

[ref91] Mußmann M. Hu F. Z. Richter M. De Beer D. Preisler A. Jørgensen B. B.. (2007). Insights into the genome of large sulfur bacteria revealed by analysis of single filaments. PLoS Biol. 5:e230. doi: 10.1371/journal.pbio.0050230, PMID: 17760503 PMC1951784

[ref92] Naito T. Kusano K. Kobayashi I. (1995). Selfish behavior of restriction-modification systems. Science 267, 897–899. doi: 10.1126/science.7846533 7846533

[ref93] Nakamura N. Lin H. C. Mcsweeney C. S. Mackie R. I. Gaskins H. R. (2010). Mechanisms of microbial hydrogen disposal in the human colon and implications for health and disease. Annu. Rev. Food Sci. Technol. 1, 363–395. doi: 10.1146/annurev.food.102308.124101 22129341

[ref94] Nakamura A. M. Nascimento A. S. Polikarpov I. (2017). Structural diversity of carbohydrate esterases. Biotechnol. Res. Innov. 1, 35–51. doi: 10.1016/j.biori.2017.02.001

[ref95] Neukirchen S. Sousa F. L. (2021). DiSCo: a sequence-based type-specific predictor of Dsr-dependent dissimilatory Sulphur metabolism in microbial data. Microbial Genomics 7:000603. doi: 10.1099/mgen.0.000603 34241589 PMC8477390

[ref96] Ng F. Kittelmann S. Patchett M. L. Attwood G. T. Janssen P. H. Rakonjac J.. (2016). An adhesin from hydrogen-utilizing rumen methanogen *Methanobrevibacter ruminantium* M1 binds a broad range of hydrogen-producing microorganisms. Environ. Microbiol. 18, 3010–3021. doi: 10.1111/1462-2920.13155 26643468

[ref97] O’Leary N. A. Wright M. W. Brister J. R. Ciufo S. Haddad D. Mcveigh R.. (2016). Reference sequence (RefSeq) database at NCBI: current status, taxonomic expansion, and functional annotation. Nucleic Acids Res. 44, D733–D745. doi: 10.1093/nar/gkv1189 26553804 PMC4702849

[ref98] Obradors N. Badía J. Baldomà L. Aguilar J. (1988). Anaerobic metabolism of the L-rhamnose fermentation product 1,2-propanediol in *Salmonella typhimurium*. J. Bacteriol. 170, 2159–2162. doi: 10.1128/jb.170.5.2159-2162.1988 3283105 PMC211101

[ref99] Parks D. H. Chuvochina M. Chaumeil P.-A. Rinke C. Mussig A. J. Hugenholtz P. (2020). A complete domain-to-species taxonomy for Bacteria and Archaea. Nat. Biotechnol. 38, 1079–1086. doi: 10.1038/s41587-020-0501-8 32341564

[ref100] Parks D. H. Chuvochina M. Rinke C. Mussig A. J. Chaumeil P.-A. Hugenholtz P. (2022). GTDB: an ongoing census of bacterial and archaeal diversity through a phylogenetically consistent, rank normalized and complete genome-based taxonomy. Nucleic Acids Res. 50, D785–D794. doi: 10.1093/nar/gkab776 34520557 PMC8728215

[ref101] Parks D. H. Chuvochina M. Waite D. W. Rinke C. Skarshewski A. Chaumeil P.-A.. (2018). A standardized bacterial taxonomy based on genome phylogeny substantially revises the tree of life. Nat. Biotechnol. 36, 996–1004. doi: 10.1038/nbt.4229 30148503

[ref102] Parks D. H. Imelfort M. Skennerton C. T. Hugenholtz P. Tyson G. W. (2015). CheckM: assessing the quality of microbial genomes recovered from isolates, single cells, and metagenomes. Genome Res. 25, 1043–1055. doi: 10.1101/gr.186072.114 25977477 PMC4484387

[ref103] Pascucci T. Colamartino M. Fiori E. Sacco R. Coviello A. Ventura R.. (2020). P-cresol alters brain dopamine metabolism and exacerbates autism-like behaviors in the BTBR mouse. Brain Sci. 10:233. doi: 10.3390/brainsci10040233 32294927 PMC7226382

[ref104] Peck S. C. Denger K. Burrichter A. Irwin S. M. Balskus E. P. Schleheck D. (2019). A glycyl radical enzyme enables hydrogen sulfide production by the human intestinal bacterium *Bilophila wadsworthia*. Proc. Natl. Acad. Sci. 116, 3171–3176. doi: 10.1073/pnas.1815661116 30718429 PMC6386719

[ref105] Pérez-Cobas A. E. Maiques E. Angelova A. Carrasco P. Moya A. Latorre A. (2015). Diet shapes the gut microbiota of the omnivorous cockroach *Blattella germanica*. FEMS Microbiol. Ecol. 91:fiv022. doi: 10.1093/femsec/fiv022 25764470

[ref106] Pester M. Brune A. (2007). Hydrogen is the central free intermediate during lignocellulose degradation by termite gut symbionts. ISME J. 1, 551–565. doi: 10.1038/ismej.2007.62 18043656

[ref107] Petit E. Latouf W. G. Coppi M. V. Warnick T. A. Currie D. Romashko I.. (2013). Involvement of a bacterial microcompartment in the metabolism of fucose and rhamnose by *Clostridium phytofermentans*. PLoS One 8:e54337. doi: 10.1371/journal.pone.0054337 23382892 PMC3557285

[ref108] Pruesse E. Peplies J. Glöckner F. O. (2012). SINA: accurate high-throughput multiple sequence alignment of ribosomal RNA genes. Bioinformatics 28, 1823–1829. doi: 10.1093/bioinformatics/bts252 22556368 PMC3389763

[ref109] Quast C. Pruesse E. Yilmaz P. Gerken J. Schweer T. Yarza P.. (2012). The SILVA ribosomal RNA gene database project: improved data processing and web-based tools. Nucleic Acids Res. 41, D590–D596. doi: 10.1093/nar/gks1219 23193283 PMC3531112

[ref110] Rakoff-Nahoum S. Coyne M. J. Comstock L. E. (2014). An ecological network of polysaccharide utilization among human intestinal symbionts. Curr. Biol. 24, 40–49. doi: 10.1016/j.cub.2013.10.077 24332541 PMC3924574

[ref111] Ray J. Keller K. Catena M. Juba T. Zemla M. Rajeev L.. (2014). Exploring the role of Chea3 in *Desulfovibrio vulgaris* Hildenborough motility. Front. Microbiol. 5:77. doi: 10.3389/fmicb.2014.00077 24639670 PMC3944678

[ref112] Rea S. Bowman J. P. Popovski S. Pimm C. Wright A.-D. G. (2007). *Methanobrevibacter millerae* sp. nov. and *Methanobrevibacter olleyae* sp. nov., methanogens from the ovine and bovine rumen that can utilize formate for growth. Int. J. Syst. Evol. Microbiol. 57, 450–456. doi: 10.1099/ijs.0.63984-0, PMID: 17329767

[ref113] Rey F. E. Faith J. J. Bain J. Muehlbauer M. J. Stevens R. D. Newgard C. B.. (2010). Dissecting the in vivo metabolic potential of two human gut acetogens. J. Biol. Chem. 285, 22082–22090. doi: 10.1074/jbc.m110.117713 20444704 PMC2903421

[ref114] Rey F. E. Gonzalez M. D. Cheng J. Wu M. Ahern P. P. Gordon J. I. (2013). Metabolic niche of a prominent sulfate-reducing human gut bacterium. Proc. Natl. Acad. Sci. 110, 13582–13587. doi: 10.1073/pnas.1312524110 23898195 PMC3746858

[ref115] Rinke C. Chuvochina M. Mussig A. J. Chaumeil P.-A. Davín A. A. Waite D. W.. (2021). A standardized archaeal taxonomy for the genome taxonomy database. Nat. Microbiol. 6, 946–959. doi: 10.1038/s41564-021-00918-8 34155373

[ref116] Rosenthal A. Z. Zhang X. Lucey K. S. Ottesen E. A. Trivedi V. Choi H. M. T.. (2013). Localizing transcripts to single cells suggests an important role of uncultured Deltaproteobacteria in the termite gut hydrogen economy. Proc. Natl. Acad. Sci. 110, 16163–16168. doi: 10.1073/pnas.1307876110 24043823 PMC3791709

[ref117] Saengkerdsub S. Ricke S. C. (2014). Ecology and characteristics of methanogenic archaea in animals and humans. Crit. Rev. Microbiol. 40, 97–116. doi: 10.3109/1040841x.2013.763220 23425063

[ref118] Saier M. H. Reddy V. S. Moreno-Hagelsieb G. Hendargo K. J. Zhang Y. Iddamsetty V.. (2021). The transporter classification database (TCDB): 2021 update. Nucleic Acids Res. 49, D461–D467. doi: 10.1093/nar/gkaa1004 33170213 PMC7778945

[ref119] Sakuma M. Fukami H. (1990). The aggregation pheromone of the German cockroach, *Blattella germanica* (L.) (Dictyoptera: Blattellidae): isolation and identification of the attractant components of the pheromone. Appl. Entomol. Zool. 25, 355–368. doi: 10.1303/aez.25.355

[ref120] Salyers A. A. Vercellotti J. R. West S. E. Wilkins T. D. (1977). Fermentation of mucin and plant polysaccharides by strains of Bacteroides from the human colon. Appl. Environ. Microbiol. 33, 319–322. doi: 10.1128/aem.33.2.319-322.1977 848954 PMC170684

[ref121] Samuel B. S. Hansen E. E. Manchester J. K. Coutinho P. M. Henrissat B. Fulton R.. (2007). Genomic and metabolic adaptations of *Methanobrevibacter smithii* to the human gut. Proc. Natl. Acad. Sci. 104, 10643–10648. doi: 10.1073/pnas.0704189104, PMID: 17563350 PMC1890564

[ref122] Savage K. N. Krumholz L. R. Gieg L. M. Parisi V. A. Suflita J. M. Allen J.. (2010). Biodegradation of low-molecular-weight alkanes under mesophilic, sulfate-reducing conditions: metabolic intermediates and community patterns. FEMS Microbiol. Ecol. 72, 485–495. doi: 10.1111/j.1574-6941.2010.00866.x 20402777

[ref123] Savant D. V. Shouche Y. S. Prakash S. Ranade D. R. (2002). *Methanobrevibacter acididurans* sp. nov., a novel methanogen from a sour anaerobic digester. Int. J. Syst. Evol. Microbiol. 52, 1081–1087. doi: 10.1099/00207713-52-4-1081, PMID: 12148611

[ref124] Schauer C. Thompson C. L. Brune A. (2012). The bacterial community in the gut of the cockroach *Shelfordella lateralis* reflects the close evolutionary relatedness of cockroaches and termites. Appl Environ Microbiol. 78, 2758–2767. doi: 10.1128/aem.07788-11 22327579 PMC3318830

[ref125] Schauer C. Thompson C. Brune A. (2014). Pyrotag sequencing of the gut microbiota of the cockroach *Shelfordella lateralis* reveals a highly dynamic core but only limited effects of diet on community structure. PLoS One 9:e85861. doi: 10.1371/journal.pone.0085861 24454939 PMC3893267

[ref126] Scheurwater E. Reid C. W. Clarke A. J. (2008). Lytic transglycosylases: bacterial space-making autolysins. Int. J. Biochem. Cell Biol. 40, 586–591. doi: 10.1016/j.biocel.2007.03.018 17468031

[ref127] Seemann T. (2014). Prokka: rapid prokaryotic genome annotation. Bioinformatics 30, 2068–2069. doi: 10.1093/bioinformatics/btu153 24642063

[ref128] Sehlmeyer S. Wang L. Langel D. Heckel D. G. Mohagheghi H. Petschenka G.. (2010). Flavin-dependent monooxygenases as a detoxification mechanism in insects: new insights from the arctiids (Lepidoptera). PLoS One 5:e10435. doi: 10.1371/journal.pone.0010435 20454663 PMC2862711

[ref129] Shiomi A. Nagao K. Yokota N. Tsuchiya M. Kato U. Juni N.. (2021). Extreme deformability of insect cell membranes is governed by phospholipid scrambling. Cell Rep. 35:109219. doi: 10.1016/j.celrep.2021.109219 34107250

[ref130] Shrivastava A. Patel V. K. Tang Y. Yost S. C. Dewhirst F. E. Berg H. C. (2018). Cargo transport shapes the spatial organization of a microbial community. Proc. Natl. Acad. Sci. 115, 8633–8638. doi: 10.1073/pnas.1808966115 30082394 PMC6112710

[ref131] Song Y. Lee J. S. Shin J. Lee G. M. Jin S. Kang S.. (2020). Functional cooperation of the glycine synthase-reductase and wood–Ljungdahl pathways for autotrophic growth of *Clostridium drakei*. Proc. Natl. Acad. Sci. 117, 7516–7523. doi: 10.1073/pnas.1912289117 32170009 PMC7132306

[ref132] Sprenger W. W. Hackstein J. H. P. Keltjens J. T. (2005). The energy metabolism of *Methanomicrococcus blatticola*: physiological and biochemical aspects. Antonie Van Leeuwenhoek 87, 289–299. doi: 10.1007/s10482-004-5941-5 15928982

[ref133] Sprenger W. W. Van Belzen M. C. Rosenberg J. Hackstein J. H. Keltjens J. T. (2000). *Methanomicrococcus blatticola* gen. Nov., sp. nov., a methanol-and methylamine-reducing methanogen from the hindgut of the cockroach *Periplaneta americana*. Int. J. Syst. Evol. Microbiol. 50, 1989–1999. doi: 10.1099/00207713-50-6-1989 11155972

[ref134] Stamatakis A. (2014). RAxML version 8: a tool for phylogenetic analysis and post-analysis of large phylogenies. Bioinformatics 30, 1312–1313. doi: 10.1093/bioinformatics/btu033 24451623 PMC3998144

[ref135] Stams F. J. Veenhuis M. Weenk G. H. Hansen T. A. (1983). Occurrence of polyglucose as a storage polymer in *Desulfovibrio* species and *Desulfobulbus propionicus*. Arch. Microbiol. 136, 54–59.

[ref136] Stepanauskas R. Fergusson E. A. Brown J. Poulton N. J. Tupper B. Labonté J. M.. (2017). Improved genome recovery and integrated cell-size analyses of individual uncultured microbial cells and viral particles. Nat. Commun. 8:84. doi: 10.1038/s41467-017-00128-z, PMID: 28729688 PMC5519541

[ref137] Stewart R. D. Auffret M. D. Roehe R. Watson M. (2018). Open prediction of polysaccharide utilisation loci (PUL) in 5414 public Bacteroidetes genomes using PULpy. Edinburgh, UK: Cold Spring Harbor Laboratory.

[ref138] Sutter M. Kerfeld C. A. (2022). BMC caller: a webtool to identify and analyze bacterial microcompartment types in sequence data. Biol. Direct 17:9. doi: 10.1186/s13062-022-00323-z 35484563 PMC9052549

[ref139] Sutter M. Melnicki M. R. Schulz F. Woyke T. Kerfeld C. A. (2021). A catalog of the diversity and ubiquity of bacterial microcompartments. Nat. Commun. 12:3809. doi: 10.1038/s41467-021-24126-4 34155212 PMC8217296

[ref140] Talens-Perales D. Górska A. Huson D. H. Polaina J. Marín-Navarro J. (2016). Analysis of domain architecture and phylogenetics of family 2 glycoside hydrolases (GH2). PLoS One 11:e0168035. doi: 10.1371/journal.pone.0168035 27930742 PMC5145203

[ref141] Tao X. Li Y. Huang H. Chen Y. Liu P. Li X. (2014). *Desulfovibrio vulgaris* Hildenborough prefers lactate over hydrogen as electron donor. Ann. Microbiol. 64, 451–457. doi: 10.1007/s13213-013-0675-0

[ref142] Thauer R. K. Jungermann K. Decker K. (1977). Energy conservation in chemotrophic anaerobic bacteria. Bacteriol. Rev. 41, 100–180. doi: 10.1128/br.41.1.100-180.1977 860983 PMC413997

[ref143] Thomas C. M. Taib N. Gribaldo S. Borrel G. (2021). Comparative genomic analysis of *Methanimicrococcus blatticola* provides insights into host adaptation in archaea and the evolution of methanogenesis. ISME Commun. 1:47. doi: 10.1038/s43705-021-00050-y PMC9723798 37938279

[ref144] Tinker K. A. Ottesen E. A. (2016). The core gut microbiome of the American cockroach, *Periplaneta americana*, is stable and resilient to dietary shifts. Appl. Environ. Microbiol. 82, 6603–6610. doi: 10.1128/AEM.01837-16 27590811 PMC5086554

[ref145] Tinker K. A. Ottesen E. A. (2020). Phylosymbiosis across deeply diverging lineages of omnivorous cockroaches (order Blattodea). Appl. Environ. Microbiol. 86, e02513–e02519. doi: 10.1128/AEM.02513-19 31953337 PMC7082566

[ref146] Tokura M. Ohkuma M. Kudo T. (2000). Molecular phylogeny of methanogens associated with flagellated protists in the gut and with the gut epithelium of termites. FEMS Microbiol. Ecol. 33, 233–240. doi: 10.1111/j.1574-6941.2000.tb00745.x 11098074

[ref147] Trischler R. Roth J. Sorbara M. T. Schlegel X. Müller V. (2022). A functional wood–Ljungdahl pathway devoid of a formate dehydrogenase in the gut acetogens *Blautia wexlerae*, *Blautia luti* and beyond. Environ. Microbiol. 24, 3111–3123. doi: 10.1111/1462-2920.16029 35466558

[ref148] Ueda O. Wexler H. M. Hirai K. Shibata Y. Yoshimura F. Fujimura S. (2005). Sixteen homologs of the Mex-type multidrug resistance efflux pump in *Bacteroides fragilis*. Antimicrob. Agents Chemother. 49, 2807–2815. doi: 10.1128/AAC.49.7.2807-2815.2005 15980353 PMC1168660

[ref150] Venceslau S. S. Stockdreher Y. Dahl C. Pereira I. A. C. (2014). The bacterial heterodisulfide DsrC is a key protein in dissimilatory sulfur metabolism. Biochimica et Biophysica Acta (BBA). Bioenergetics 1837, 1148–1164. doi: 10.1016/j.bbabio.2014.03.007, PMID: 24662917

[ref151] Vera-Ponce De León A. Jahnes B. C. Duan J. Camuy-Vélez L. A. Sabree Z. L. (2020). Cultivable, host-specific bacteroidetes symbionts exhibit diverse polysaccharolytic strategies. Appl. Environ. Microbiol. 86:e00091–20. doi: 10.1128/aem.00091-20 32060023 PMC7117922

[ref152] Vera-Ponce De Leon A. Schneider M. G. Jahnes B. C. Sadowski V. Camuy-Vélez L. A. Duan J.. (2022). Genetic drift and host-adaptive features likely underlie the cladogenesis of insect-associated Lachnospiraceae. Genome Biol. Evol. 14:evac086. doi: 10.1093/gbe/evac086 35679131 PMC9210297

[ref153] Větrovský T. Baldrian P. (2013). The variability of the 16S rRNA gene in bacterial genomes and its consequences for bacterial community analyses. PLoS One 8:e57923. doi: 10.1371/journal.pone.0057923 23460914 PMC3583900

[ref154] Waite D. W. Chuvochina M. Pelikan C. Parks D. H. Yilmaz P. Wagner M.. (2020). Proposal to reclassify the proteobacterial classes Deltaproteobacteria and Oligoflexia, and the phylum Thermodesulfobacteria into four phyla reflecting major functional capabilities. Int. J. Syst. Evol. Microbiol. 70, 5972–6016. doi: 10.1099/ijsem.0.004213 33151140

[ref155] Wang Z. Klipfell E. Bennett B. J. Koeth R. Levison B. S. Dugar B.. (2011). Gut flora metabolism of phosphatidylcholine promotes cardiovascular disease. Nature 472, 57–63. doi: 10.1038/nature09922 21475195 PMC3086762

[ref156] Wang S. P. Rubio L. A. Duncan S. H. Donachie G. E. Holtrop G. Lo G.. (2020). Pivotal roles for pH, lactate, and lactate-utilizing bacteria in the stability of a human colonic microbial ecosystem. mSystems 5, e00645–e00620. doi: 10.1128/mSystems.00645-20 PMC7483512 32900872

[ref157] Westram R. Bader K. Prüße E. Kumar Y. Meier H. Glöckner F.O.. (2004). ARB: a software environment for sequence data, In Handbook of molecular microbial ecology i: Metagenomics and complementary approaches, ed. Bruijn F. De, 399–406.

[ref158] Wirth J. S. Bush E. C. (2023). Automating microbial taxonomy workflows with PHANTASM: Phylogenomic analyses for the taxonomy and systematics of microbes. Nucleic Acids Res. 51, 3067–3077. doi: 10.1093/nar/gkad196 36938868 PMC10123096

[ref159] Woyke T. Xie G. Copeland A. González J. M. Han C. Kiss H.. (2009). Assembling the marine metagenome, one cell at a time. PLoS One 4:e5299. doi: 10.1371/journal.pone.0005299, PMID: 19390573 PMC2668756

[ref160] Xing M. Wei Y. Zhou Y. Zhang J. Lin L. Hu Y.. (2019). Radical-mediated C-S bond cleavage in C2 sulfonate degradation by anaerobic bacteria. Nat. Commun. 10:1609. doi: 10.1038/s41467-019-09618-8, PMID: 30962433 PMC6453916

[ref161] Yuki M. Kuwahara H. Shintani M. Izawa K. Sato T. Starns D.. (2015). Dominant ectosymbiotic bacteria of cellulolytic protists in the termite gut also have the potential to digest lignocellulose. Environ. Microbiol. 17, 4942–4953. doi: 10.1111/1462-2920.12945 26079531

[ref162] Zane G. M. Yen H.-C. B. Wall J. D. (2010). Effect of the deletion of qmoABC and the promoter-distal gene encoding a hypothetical protein on sulfate reduction in *Desulfovibrio vulgaris* Hildenborough. Appl. Environ. Microbiol. 76, 5500–5509. doi: 10.1128/aem.00691-10 20581180 PMC2918943

[ref163] Zbell A. L. Maier R. J. (2009). Role of the Hya hydrogenase in recycling of anaerobically produced H_2_ in *Salmonella enterica* serovar typhimurium. Appl. Environ. Microbiol. 75, 1456–1459. doi: 10.1128/AEM.02064-08 19114523 PMC2648180

