# Supplementary material for: Disentangling hindgut metabolism in the American cockroach through single-cell genomics and metatranscriptomics
Source: Front Microbiol. 2023 May 30;14:1156809. doi: 10.3389/fmicb.2023.1156809 (PMC10266427; doi:10.3389/fmicb.2023.1156809)
Supplement: Supplementary file 5 [file Data_Sheet_1.PDF]

Supplementary Material

Disentangling hindgut metabolism in the American cockroach through single-cell genomics and metatranscriptomics

Helen E. Dukes[1], Kara A. Tinker[2], Elizabeth A. Ottesen[1*]

*** Correspondence:** Dr. Elizabeth Ottesen: ottesen@uga.edu

Contents

1. Supplementary Methods 2
2. Supplementary Tables
   Descriptions of supplementary tables 1-4 (uploaded as separate files) 6
   Supplementary Table 5. Information on genes identified via HMMer 7
   Supplementary Table 6. Nutrient information for 100g serving of diet treatment 8
3. Supplementary Figures
   Supplementary Figure 1. PHANTASM tree for *Synergistota* D05. 9
   Supplementary Figure 2. PHANTASM tree for *Clostridia* SAG F22. 10
   Supplementary Figure 3. PHANTASM tree for *Ruminococcaceae* SAG O13. 11
   Supplementary Figure 4. *Bacteroidota* genome tree built from single-copy genes identified in SAGs with CheckM. 12
   Supplementary Figure 5. *Firmicutes* genome tree built from single-copy genes identified in SAGs with CheckM. 13
   Supplementary Figure 6. *Desulfobacterota* genome tree built from single-copy genes identified in SAGs with CheckM. 14
   Supplementary Figure 7. 16S phylogenetic tree for *Synergistota*. 15
3. References cited in Supplementary Materials 16

1 Supplementary Methods

1.1 Cockroach Hindgut Sample Collection

Metagenomic and metatranscriptomic data that was collected as part of an experiment examining cockroach gut microbial responses to a diverse set of dietary compositions was used to evaluate the abundance and transcriptional activity of our SAGs. As our aim in this study was to generate an overall picture of microbial abundance and activity, cross-cutting averages across all samples and conditions are presented presented throughout the text.

Laboratory cultures of *P. americana* are maintained in mixed age, mixed sex colonies in aquarium tanks at room temperature on a diet of dog food *ad libitum*. Each culture tank is provided with corn cob bedding, cardboard tubes for nesting, and a cellulose sponge saturated with water. For this experiment, 25 adult cockroaches per treatment group were selected from the culture tanks, weighed, and marked for later identification. Each dietary treatment group was housed in a single plastic tank that contained pebbles for bedding, a large weigh boat for shelter, and food and water in weigh boats. Food, water, and weigh boats were changed as needed, and any ootheca or deceased cockroaches were removed daily. Treatments included dog food (Kroger Nutritionally Complete Bite Size Adult Dog Food; composed of 21% protein, 8% fat, and 6% fiber), bran (Bob's Red Mill Organic High Fiber Oat Bran Hot Cereal), butter (Kroger Unsalted Butter Sticks), tuna (StarKist Selects Low Sodium Chunk Light Tuna in Water), and starvation (Table S3).

After 14 days on the assigned diet, all cockroaches were sacrificed and their hindgut microbiota preserved for DNA (9 hindguts) or RNA extraction (16 hindguts). Individual cockroaches were removed from tanks, weighed, and placed on ice in sterile culture plates. Once sufficiently torpid, cockroaches were dissected, and the entire gut was removed. Any visible debris, including fat bodies or exoskeleton, was removed with forceps. The hindgut was then separated from the rest of the gut using a scalpel and submerged in either 100 μL of 1XTE buffer or RNALater (Ambion, Austin, TX, USA). Hindgut samples placed in 1XTE buffer were immediately stored at -80°C. Hindgut samples placed in RNALater were crushed with a sterile glass stirring rod. The suspended gut lumen was then removed and stored at -80°C.

1.2 DNA Extraction

Microbial DNA was extracted from hindgut samples using a modified version of the EZNA Bacteria Kit (Omega Biotek, Norcross, GA). This protocol can be found at dx.doi.org/10.17504/protocols.io.jz5cp86. Final DNA concentrations (ranging from 30 to 1800 ng/μL) and A260/A280 were measured using a NanoDrop Lite spectrophotometer (Thermo Scientific, Wilmington, DE).

1.3 Total RNA Extraction

Initial attempts at extracting RNA from the entire preserved hindgut sample failed due to high levels of RNA degradation from host inhibitors (Figure A.1). Thus, microbial RNA was extracted from one half of the total volume (50 μL) of sample using a modified version of the HP Total RNA Kit (Omega Biotek, Norcross, GA). Find a detailed protocol here: https://dx.doi.org/10.17504/protocols.io.jz6cp9e. DNases were removed using Invitrogen's Turbo DNA-free Kit (Thermo Scientific, Wilmington, DE) before cleaning and concentrating the extracted RNA using the EZNA MicroElute RNA Clean-Up Kit (Omega Biotek, Norcross, GA).

0.1 volume 10X Turbo DNase Buffer (5µL) and 1µL TURBO DNase (as supplied by the Turbo DNA-free kit) were gently mixed into the eluted RNA before incubation at 37°C for 25 min. After DNase removal, the manufacturer's protocol for the EZNA MicroElute RNA Clean Up Kit (July 2014 version) was followed. Quality of total RNA was confirmed on the Bioanalyzer using the RNA 6000 Pico Total RNA Kit. The top four total RNA samples per dietary treatment were identified and used for rRNA depletion and library preparation (Agilent, Santa Clara, CA).

1.4 rRNA Depletion and Metatranscriptome Library Preparation

Custom biotin-labeled antisense RNA probes were made using pooled extracted microbial DNA from the hindguts of one representative cockroach per dietary treatment using a protocol adapted from (Stewart et al., 2010). First, extracted DNA was amplified using T7-appended primers that target universal archaeal, bacterial, and eukyarotic ribosomal gene regions and cockroach host tissue (Table 4.2). PCR reactions had a total volume of 50 µL and were composed of 5X Q5 buffer (NEB, Ipswitch, MA), 200 µM dNTPS, 0.5 F primer, 0.5 µM R primer, 100 ng template DNA, and 0.02 U/µL Q5 Hot Start High-Fidelity DNA polymerase (NEB). PCR conditions were 98°C for 2 min; 35 cycles at 98°C for 20 s, variable for 20 s, and 72°C for 2 min; and 72°C for 3 min.

Next, the Ampliscribe T-7 Flash Biotin-RNA Transcription Kit (Lucigen Corp., Middleton, WI) was used to complete in vitro transcription of biotin-labeled antisense RNA probes. Transcription reactions had a total volume of 10 µL and were composed of 1 µL AmpliScribe T-7 Flash 10X buffer, 4 µL NTP/Biotin-UTP premix, 100mM DDT, 0.25 µL Riboguard RNase, 1 µLAmpliScribe T-7 Flash Enzyme Solution, and 500ng of template DNA from the previous reaction. Transcription reactions were incubated at 37°C for 4 hrs. After transcription, the reactions were purified using the MEGAclear Kit Purification for Large Scale Transcription Reactions (Life Technologies, Carlsbad, CA). In brief, the reaction volume was brought to 100 µL and 350 µL of Binding Solution Concentrate (as supplied by kit) and 250 µL of 100% ethanol was added to the sample. The mixture was applied to a Filter Cartridge (as supplied by kit) before two washes with 500 µL Wash Solution (as supplied by kit). RNA was eluted into 50 µL Elution Solution (as supplied by kit) after incubation at 65°C for 5 min.

rRNA was depleted from total RNA through hybridization to custom RNA probes before removal with magnetic beads. Hybridization reactions had a total volume of 50 µL and were composed of 1 µL Superase-In RNase Inhibitor (Thermo Scientific), 2.5 µL 20X Sodium chloride-citrate (SSC) buffer, 10 µL 100% Formamide, antisense RNA probes (Table 4.3), and extracted total RNA. Reactions were incubated at 70°C for 5 min followed by a rampdown to 25°C using 5°C increments for 1 min each before incubating at room temperature for 2-5 min.

100 µL streptavidin-coated magnetic beads (NEB) per sample were prepared during the hybridization reaction. Beads were applied to a magnetic separation rack and the supernatant was removed. Beads were washed three times with an equal volume of 0.1N NaOH (first wash) or 1X SSC buffer (second and third wash), aliquoted into 100 µL volumes, and kept on ice until the hybridization reaction was complete. After completion, supernatant was removed from pre-aliquoted beads and 40 µL of 1X SSC and 10 µL 20% formamide was added to the completed hybridization reaction before applying to the mixture to the dried beads.

The mixture was incubated at room temperature for 10 min, with occasional flicking to mix. Beads were captured on a magnetic rack and supernatant containing the depleted RNA was collected in a new rube. Remaining beads were resuspended with 100 µL 1X SSC and captured on the magnetic rack. Supernatant was transferred to the new tube, for a total volume of 200 µL depleted

RNA. Depleted RNA was cleaned using the RNeasy MinElute kit (Omega) before quality confirmation, as described above.

Sequencing libraries prepared using the NEBNext Ultra Directional RNA Library Prep Kit for Illumina (NEB) following product guidelines with an RNA fragmentation time of 10 min. The prepared library was submitted to the Georgia Genomics facility for normalization, pooling, and sequencing (Illumina HiSeq; Illumina, Inc., San Diego, CA).

1.5 Metagenome Sample Collection and Library Preparation

DNA was extracted from *P. americana* hindgut contents as previously described (see https://dx.doi.org/10.17504/protocols.io.jz5cp86). This DNA was then sheared via sonication using a Diagenode BioRuptor Pico with 0.65 mL microtubes (catalog #C30010011) and adaptor. Extracted samples were diluted in TE to achieve a final volume of 100 µL at a DNA concentration of 10 ng/µL. They were vortexed well to mix, centrifuged for 10 sec, and placed on ice for 15 min before sonicating. The BioRuptor Pico was programmed for 10x cycles with each cycle consisting of 30 sec sonication ON and 30 sec sonication OFF to result in an average insert size of 300 bp. The 2100 Bioanalyzer (Agilent Technologies, Santa Clara CA, catalog #G2939A) was used with the High Sensitivity DNA Kit (Agilent Technologies, catalog #5067-4626) to confirm the quantity and size distribution of fragments while optimizing this protocol (see Figure S8 below for example).

Sheared samples were end-repaired, dA-tailed, and adaptor ligated as instructed with the NEBNext Ultra II DNA Library Prep Kit for Illumina (New England BioLabs Inc., catalog #E7645 includes #E7442 and #E7445,). Adaptor-ligated fragments were size-selected with a magnetic bead protocol from Dr. Travis Glenn at UGA (Glenn et al., 2019), derived from (Rohland and Reich, 2012). Prepared beads are tested before use to ensure that they size-select as expected. Here, beads were added to the sample at a volume ratio of 0.85 to select for fragments with an average size of 450 bp. Briefly: 0.85x volume beads was added to the adapter ligation reaction mixture, vortexed to mix, and incubated at room temperature for 5 min; tubes were then placed on a NEBNext Magnetic Separation Rack (New England BioLabs Inc., catalog #S1515S) and the supernatant removed once cleared; the beads were then washed twice with 300 µL 70% ethanol; after washing, the beads were left to dry on the magnetic rack at room temperature for 2-4 min; the DNA was eluted from the beads by resuspending them in 20 µL TE for 2 min; finally, the sample was placed back on the magnetic rack to aspirate the cleared supernatant for further processing.

Size-selected and adapter-ligated DNA was enriched via PCR using the NEBNext Ultra II DNA Library Prep Kit for Illumina with the NEBNext Multiplex Oligos for Illumina as dual index primers (New England BioLabs Inc., catalog #E7645 with #E7600). For amplification, we followed kit instructions for reagent concentrations but targeted a final volume of 25 µL instead of 50 µL. Six amplification cycles were used for PCR. PCR products were cleaned up using the beads and protocol as described above, except the ratio of beads to sample was 1:1. These prepared libraries were run on the Bioanalyzer to check for adapter dimers (80-100 bp) and primer dimers (approximately 150 bp). Samples that did not show PCR product were reamplified with 8x cycles instead of 6x as above.

Equal amounts (ng) of dual-indexed DNA were pooled together for sequencing. To concentrate this pool, it was put through a final size-selective bead cleanup (as above, 0.85x ratio) and eluted in TE using 10% of the initial pool volume.

It was found that both the Qubit Fluorometer (2.0, Invitrogen, ThermoFisher Scientific, Waltham MA, catalog #Q32866) and NanoDrop Lite Spectrophotometer (ThermoScientific, ThermoFisher Scientific, catalog #ND-LITE-PR) severely over-estimated the amount of DNA in the prepared libraries, so DNA concentration of the final pool concentration was measured on the Bioanalyzer. The final pool (3.5 nM DNA) was sent for sequencing at Novogene Corporation Inc. (Sacramento CA) following their specifications.

1.6 Metagenome and Metatranscriptome Sequence Processing

Initial quality checks were performed using FastQC and MultiQC to check for low-quality regions (https://www.bioinformatics.babraham.ac.uk/projects/fastqc/) (Ewels et al., 2016). Adapter sequences were trimmed with BBDuk from BBTools (http://sourceforge.net/projects/bbmap/; same for all following "BB" programs).

All raw reads were filtered to removed rRNA sequences with SortMeRNA (Kopylova et al., 2012). Sequences aligning to the *Periplaneta americana* genome (Li et al., 2018) or fat body endosymbiont—*Blattabacterium*—genome or plasmid (Sabree et al., 2009) were filtered out using BBSplit. Forward and reverse reads were then merged with BBMerge. Reads that did not merge properly were manually merged by reverse-complementing the reverse read and joining it to the forward read with NNNNNNNNN (interpreted by BLAST as a gap of unknown length) to simplify homology-based read mapping.

The resulting reads were mapped to single cell-amplified genomes extracted from the hindgut of a *P. americana* individual on a dog food diet and RefSeq Microbial Genomes (O'Leary et al., 2016) using DIAMOND v2.0.9 (Buchfink et al., 2015) (blastx --sensitive --top 1 --minscore 50). All hits with e-values < 10^{-10} were compared between the databases to assign hits to the best match from either source. Reads with best matches to SAGs were extracted and tallied by individual SAG. Reads with equal top hits to multiple organisms were counted as a hit to each to avoid under-counting conserved genes and SAG clusters with high ANI. Top transcribed functions were determined by sorting functions by their average relative abundance across all diets within that SAG. See main manuscript for description of mapping transcripts to functional databases KEGG, CAZy, and the Transporter Classification Database.

1.7 Acknowledgements

We thank Trace Borchardt, Dr. Morgan Teachey, and Nicollette Lewis for their assistance with initial diet experiment set up and gut sample collection. We also thank Nicollette Lewis for her assistance with DNA extractions.

2 Supplementary Figures and Tables

2.1 Tables

Supplementary Table 1 contains sheets with the following labels and contents:

- **Table_S1-1.** Descriptions for sheets in Table S1.
- **Table S1-2.** Genome Taxonomy Database taxon names used in this publication and their corresponding name, BioSample ID, and accession in NCBI.
- **Table_S1-3.** Summary statistics for all single-cell genomes.
- **Table S1-4.** Relative 16S abundances of phyla from (Tinker and Ottesen, 2016).
- **Table_S1-5.** Relative 16S abundances of families from (Tinker and Ottesen, 2016).
- **Table_S1-6.** Relative 16S abundances of genera from (Tinker and Ottesen, 2016).
- **Table_S1-7.** Average nucleotide identities between SAGs in this dataset.
- **Table_S1-8.** Metatranscriptome library size and quality control data.
- **Table_S1-9.** Metatranscriptome sequence counts and proportions matching RefSeq and SAG databases.
- **Table_S1-10.** Metagenome library size and quality control data.
- **Table_S1-11. M**etagenome sequence counts and proportions matching RefSeq and SAG databases.

Supplementary Table 2 contains sheets with the following labels and contents:

- **Table_S2-1.** Descriptions for sheets in Table S2.
- **Table S2-2.** KEGG ortholog hitcounts per single-cell genome.
- **Table_S2-3.** KEGG ortholog proportions per SAG in metagenome.
- **Table S2-4.** KEGG ortholog proportions per SAG in metatranscriptome.
- **Table_S2-5.** Accessions for KEGG ortholog (KO) hits to SAGs.

Supplementary Table 3 contains sheets with the following labels and contents:

- **Table_S3-1.** Descriptions for sheets in Table S3.
- **Table S3-2.** Transporter Classification Database ID hitcounts per single-cell genome.
- **Table_S3-3.** Transporter Classification Database ID proportions per SAG in metagenome.
- **Table S3-4.** Transporter Classification Database ID proportions per SAG in metatranscriptome.
- **Table_S3-5.** Accessions for Transporter Classification Database ID (TCID) hits to SAGs.

Supplementary Table 4 contains sheets with the following labels and contents:

- **Table_S4-1.** Descriptions for sheets in Table S4.
- **Table S4-2.** CAZyme HMM domain hitcounts per single-cell genome.
- **Table_S4-3.** CAZyme HMM domain proportions per SAG in metagenome.
- **Table S4-4.** CAZyme HMM domain proportions per SAG in metatranscriptome.
- **Table_S4-5.** Accessions for CAZyme HMM domain hits to SAGs.

Supplementary Table 5. Information on genes identified via HMMer.

Gene	Database link	Acc	Notes; domain score threshold
**Dockerin type I domain**	Pfam	PF00404	To identify cellulosome producers; domain: 22.9
**Cohesin**	Pfam	PF00963	To identify cellulosome producers; domain: 24.1
**Relaxase**	Pfam	PF03432	Domain: 24.1
**cutC** (choline trimethylamine lyase)	GenBank: ACL49259, ABB40076, EDU36695, EEU12078, EFJ62362, EEW38822, EEI47333	ACL49259, ABB40076, EDU36695, EEU12078, EFJ62362, EEW38822, EEI47333	Genes chosen using (Rath et al., 2017;Jameson et al., 2018) and aligned with (Madeira et al., 2019). Alignment was used to make an HMM profile (Eddy, 2011). Domain: 906.4 - threshold chosen based on (Rath et al., 2017)
**EutC** (Ethanolamine ammonia-lyase)	Pfam	PF05985	Domain: 25.8
**Chitinase A**	Pfam	PF08329	Domain: 21.2
**NifH** (nitrogenase)	NCBI	TIGR01282	hits in methanogens only; domain: 653.5
**pucL** (uricase)	Pfam	PF01041	none found above cut-off; domain: 19.8
**ureC** (urease)	NCBI	TIGR01792	none found above cut-off, but many labeled as amidohydrolase; domain: 680.0
**katG** (catalase)	NCBI	TIGR00198	none found above cut-off; domain: 770.3
**SOD** (superoxide dismutase)	Pfam: SOD_Cu, SOD_Fe_N, SOD_Fe_C, SOD_Ni	PF00080 (SOD_Cu), PF00081 (SOD_Fe_N), PF02777 (SOD_Fe_C), PF09055 (SOD_Ni)	no SOD_Fe(_N nor _C) found above cut-off; domains: 28.9 (SOD_Cu), 27.0 (SOD_Fe_N), 20.9 (SOD_Fe_C), 25.0 (SOD_Ni)
**Catalase_C** (C-terminal domain)	Pfam	PF18011	none found above cut-off; domain: 24.6
**peroxidase**	Pfam	PF00141	none found above cut-off; domain: 27.0

Supplementary Table 6. Nutrient information for 100g serving of diet treatment. All cockroaches were raised on our laboratory control diet of dog food, composed of 21% protein, 8% fat, and 6% fiber. Carbohydrate contents of dog food are estimated based on levels of other macronutrients, ash, and moisture.

Diet Treatment	Calories	Protein	Carbohydrate	Fat	Fiber
Bran	375	17.5	67.5	5	17.5
Butter	714	0	0	79	0
Tuna	107	27	0	1	0
Starvation	N/A	N/A	N/A	N/A	N/A
Dog Food	350-399	21	45	8	6

2.2 Supplementary Figures

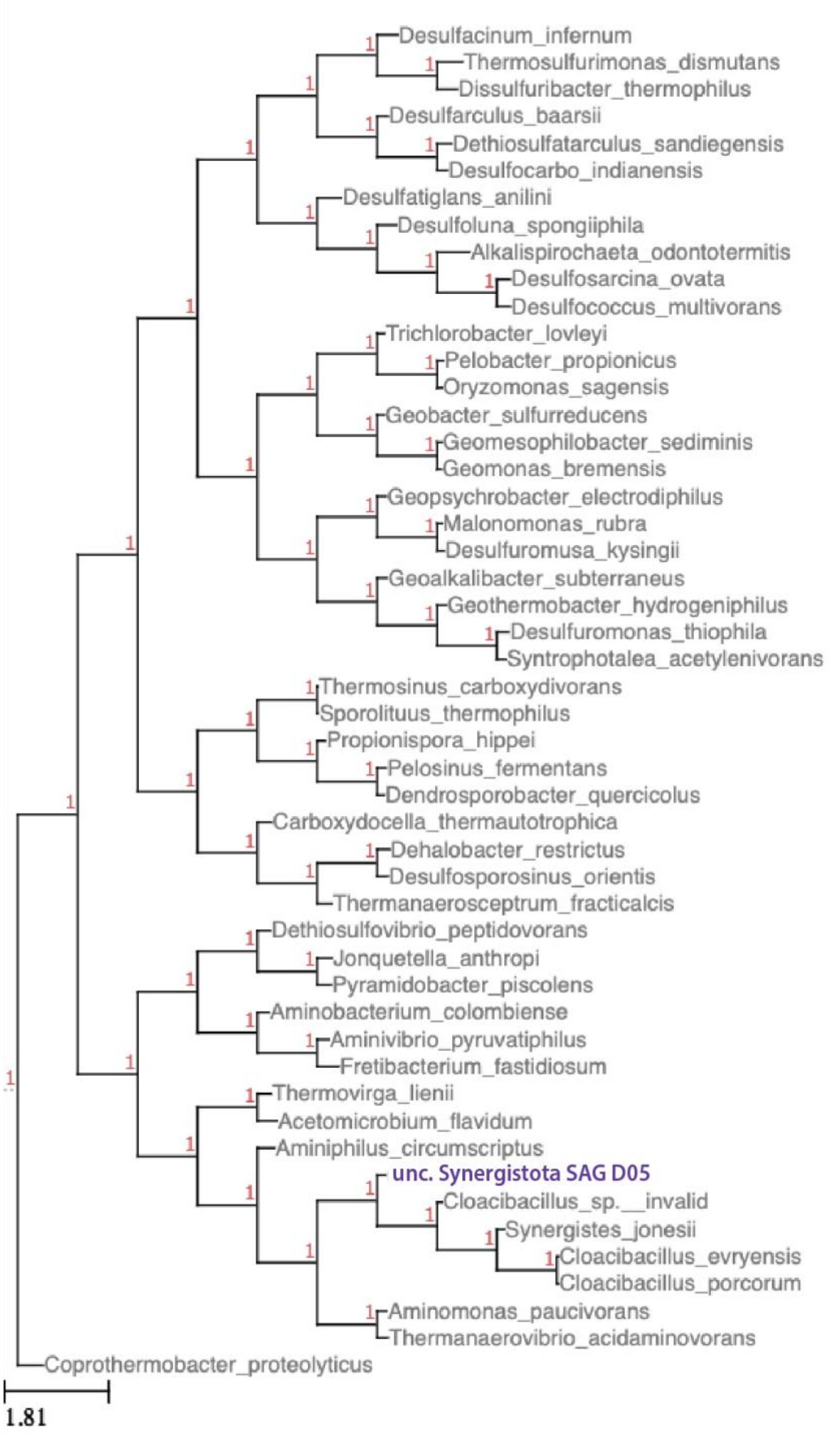

Supplementary Figure 2. PHANTASM tree for *Synergistota* D05. PHANTASM was used to identify potential marker genes for reference picking, alignment, and tree building (Wirth and Bush, 2023). Valine tRNA-ligase (*valS*) was the marker gene used here.

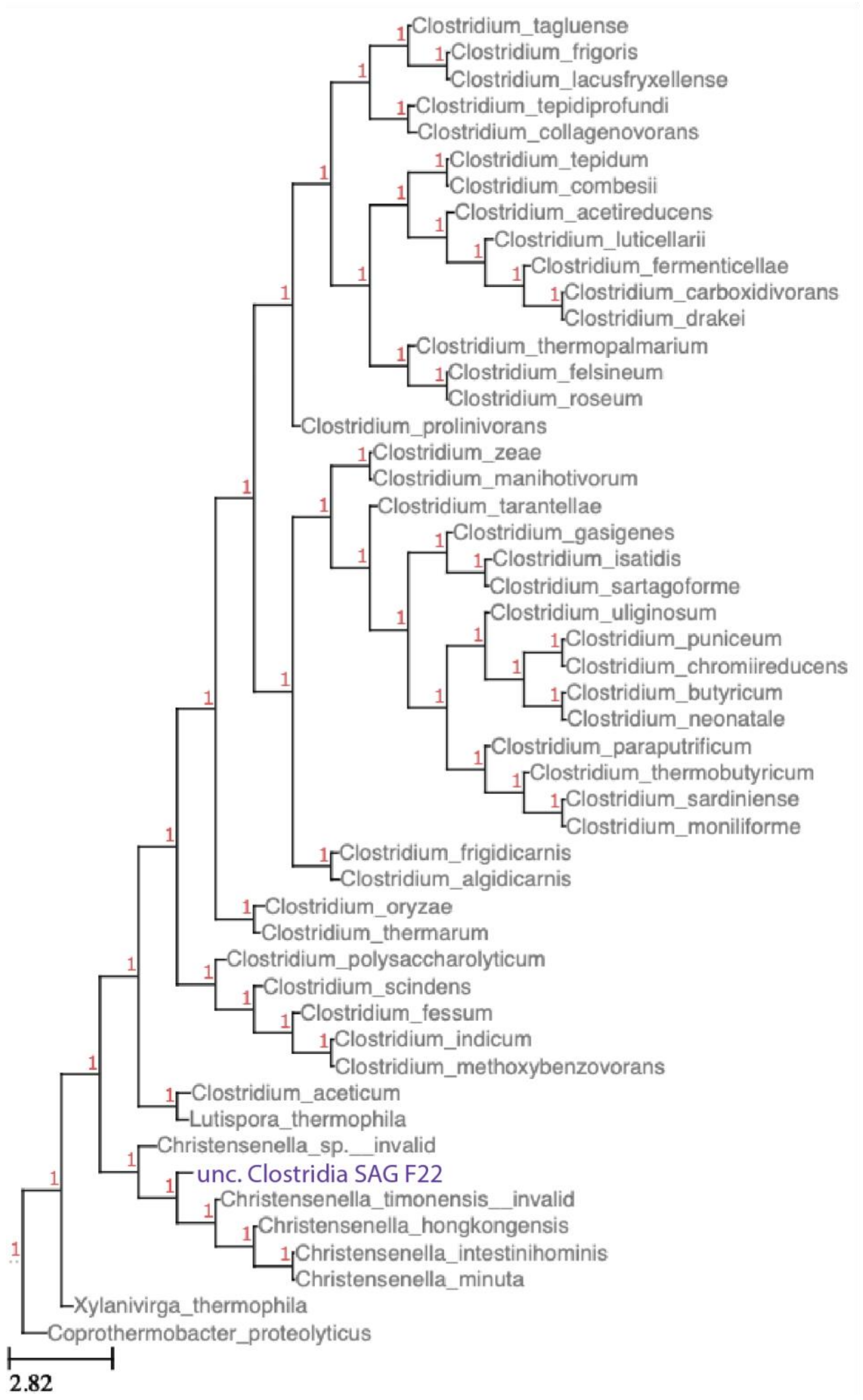

Supplementary Figure 2. PHANTASM tree for *Clostridia* SAG F22. PHANTASM was used to identify potential marker genes for reference picking, alignment, and tree building (Wirth and Bush, 2023). The 50S ribosomal protein L19 (*rplS*) was the marker gene used here.

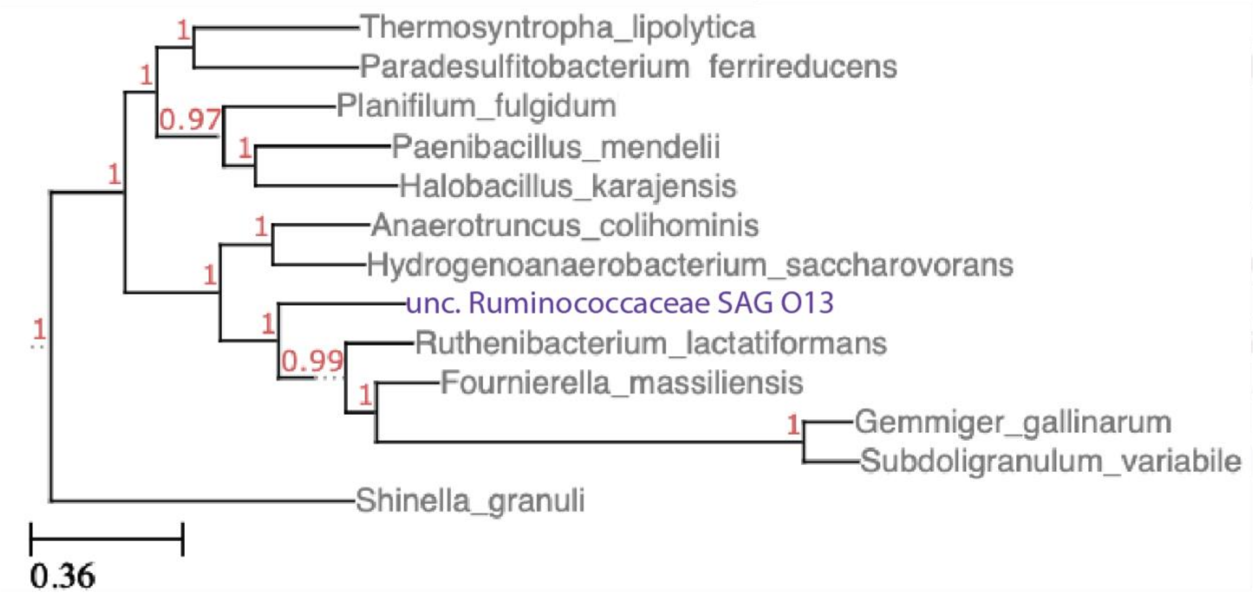

Supplementary Figure 3. PHANTASM tree for *Ruminococcaceae* SAG O13. PHANTASM was used to identify potential marker genes for reference picking, alignment, and tree building (Wirth and Bush, 2023). DNA polymerase III (*polC*) was the marker gene used here.

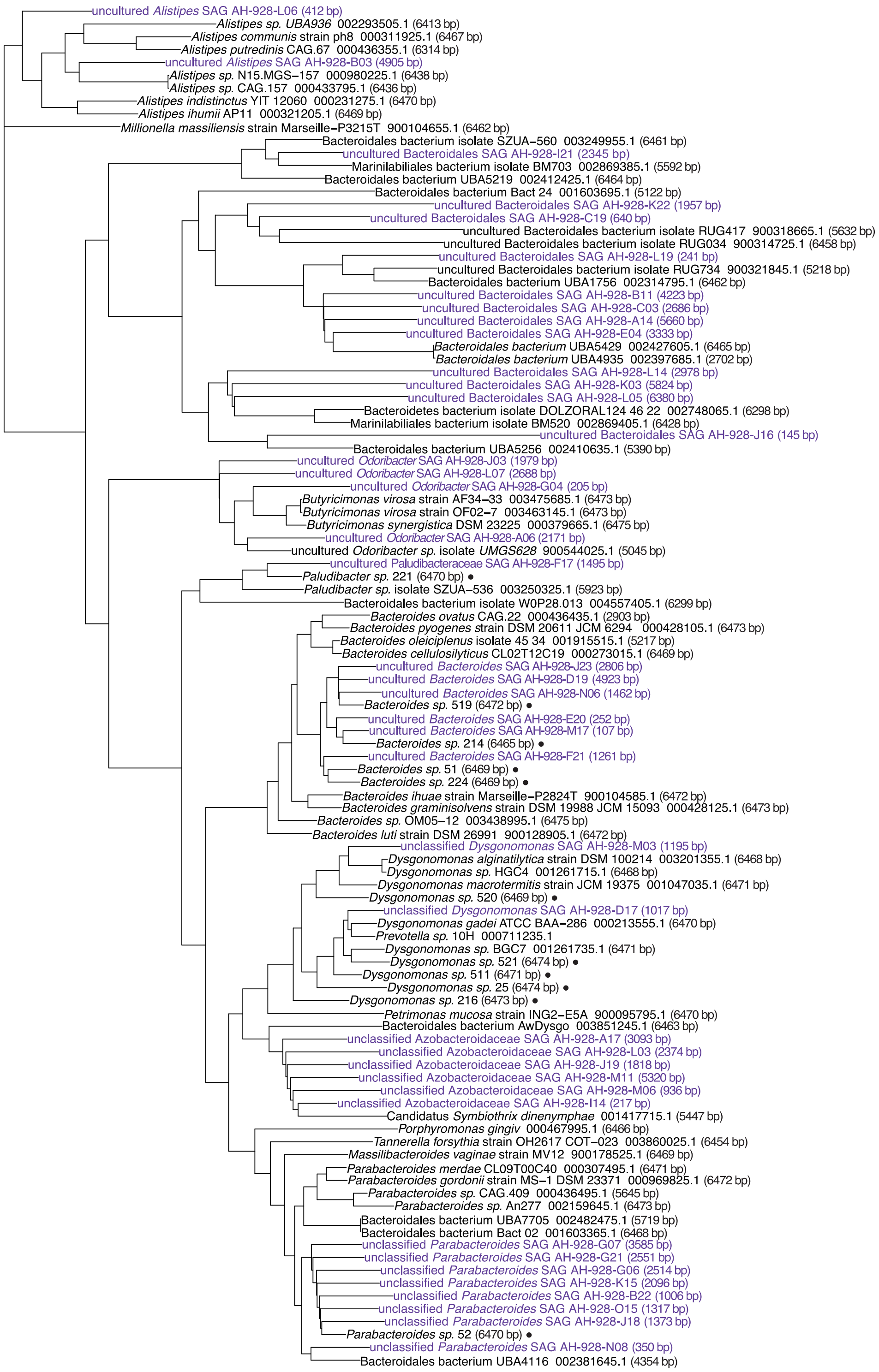

Supplementary Figure 4. *Bacteroidota* genome tree built from single-copy genes identified in SAGs with CheckM. Trees were built using reference genomes (in black), and SAGs (in purple) were placed on the reference tree. Lengths of aligned fragments are shown in parentheses. References from (Vera-Ponce De León et al., 2020) are marked with a black circle (●).

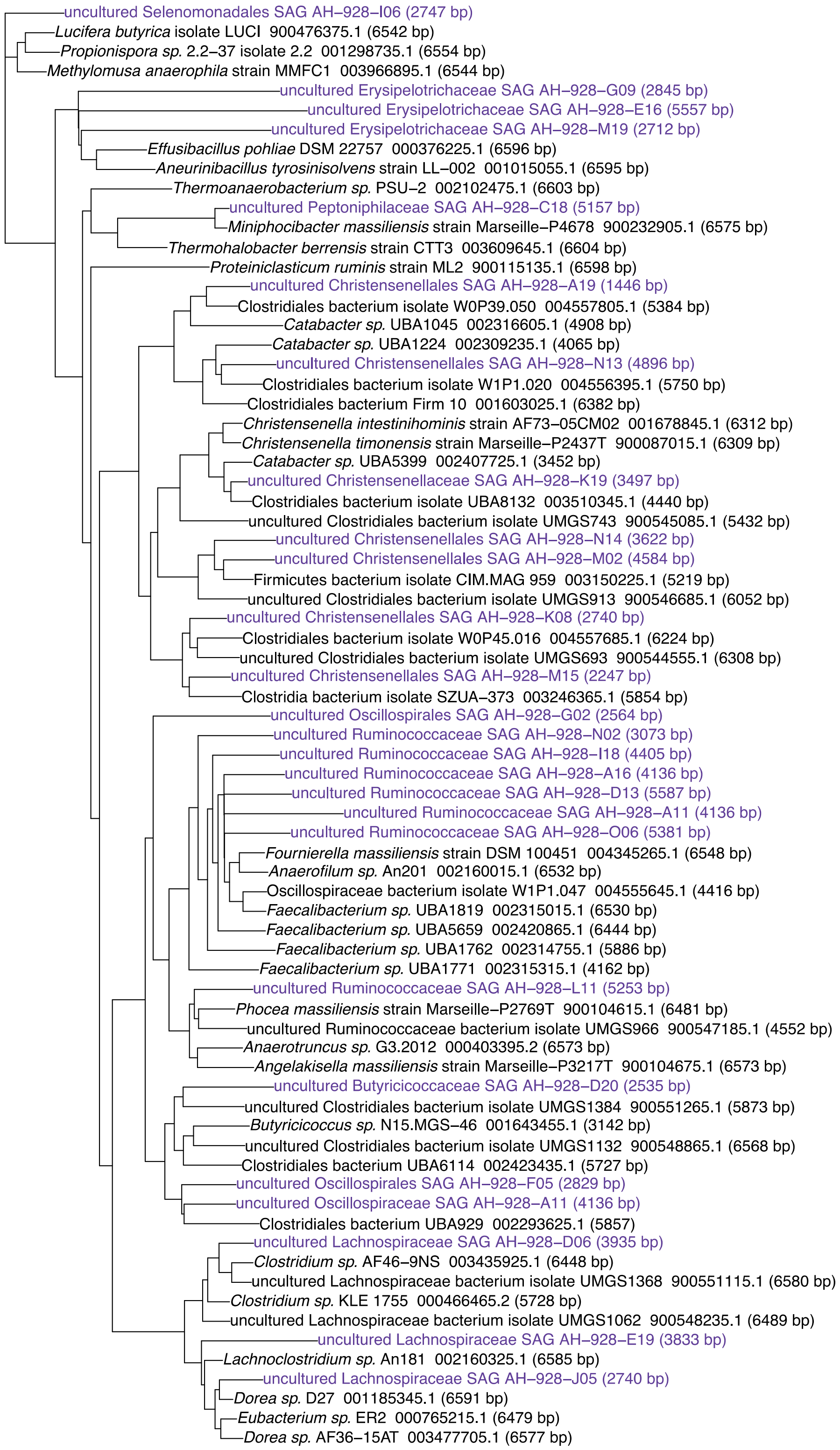

Supplementary Figure 5. *Firmicutes* genome tree built from single-copy genes identified in SAGs with CheckM. Trees were built using reference genomes (in black), and SAGs (in purple) were placed on the reference tree. Lengths of aligned fragments are shown in parentheses.

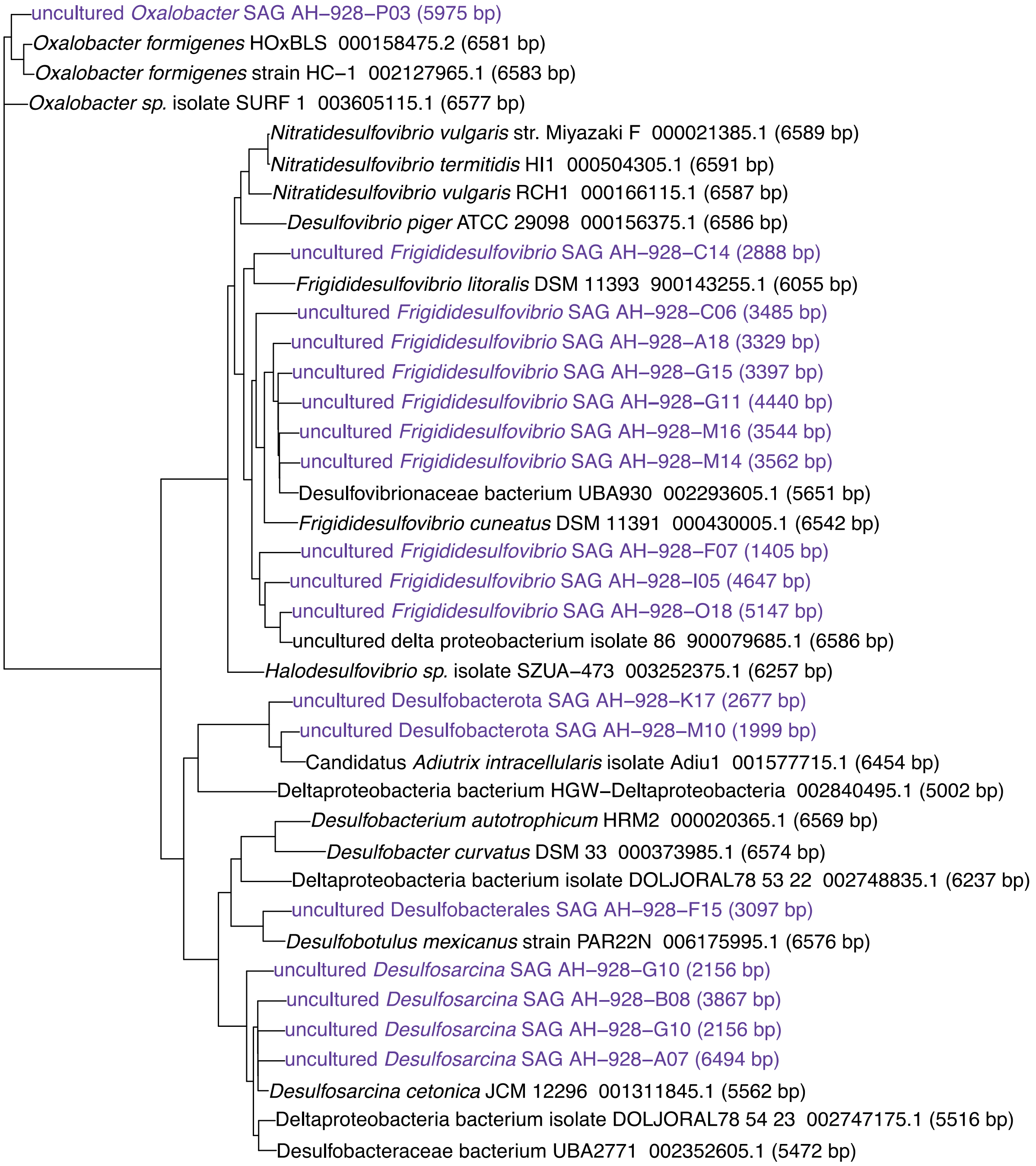

Supplementary Figure 6. *Desulfobacterota* genome tree built from single-copy genes identified in SAGs with CheckM. Trees were built using reference genomes (in black), and SAGs (in purple) were placed on the reference tree. Lengths of aligned fragments are shown in parentheses.

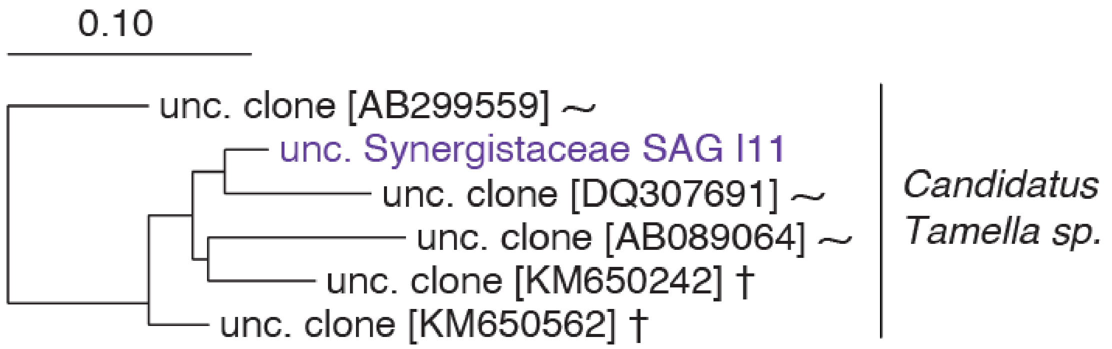

Supplementary Figure 7. 16S phylogenetic tree for *Synergistota*. The only SAG from this phylum with a recovered 16S rRNA gene was *Synergistaceae* SAG I11 (in purple). The alignment length was 1450 bp after filtering positions with majority gaps. Symbols for references recovered from environmental sources: *P. americana* and other cockroaches (†), termites and other insects (~). SILVA accessions are found in brackets.

3 References Cited in Supplementary Materials

Buchfink, B., Xie, C., and Huson, D.H. (2015). Fast and sensitive protein alignment using diamond. *Nature Methods* 12**,** 59-60. 10.1038/nmeth.3176

Eddy, S.R. (2011). Accelerated profile hmm searches. *PLoS Computational Biology* 7**,** e1002195. 10.1371/journal.pcbi.1002195

Ewels, P., Magnusson, M., Lundin, S., and Käller, M. (2016). Multiqc: Summarize analysis results for multiple tools and samples in a single report. *Bioinformatics* 32**,** 3047-3048. 10.1093/bioinformatics/btw354

Glenn, T.C., Nilsen, R.A., Kieran, T.J., Sanders, J.G., Bayona-Vásquez, N.J., Finger, J.W., et al. (2019). Adapterama i: Universal stubs and primers for 384 unique dual-indexed or 147,456 combinatorially-indexed illumina libraries (itru & inext). *PeerJ* 7**,** e7755. 10.7717/peerj.7755

Jameson, E., Quareshy, M., and Chen, Y. (2018). Methodological considerations for the identification of choline and carnitine-degrading bacteria in the gut. *Methods* 149**,** 42-48.

Kopylova, E., Noé, L., and Touzet, H. (2012). Sortmerna: Fast and accurate filtering of ribosomal rnas in metatranscriptomic data. *Bioinformatics* 28**,** 3211-3217. 10.1093/bioinformatics/bts611

Li, S., Zhu, S., Jia, Q., Yuan, D., Ren, C., Li, K., et al. (2018). The genomic and functional landscapes of developmental plasticity in the american cockroach. *Nature Communications* 9. 10.1038/s41467-018-03281-1

Madeira, F., Park, Y.M., Lee, J., Buso, N., Gur, T., Madhusoodanan, N., et al. (2019). The embl-ebi search and sequence analysis tools apis in 2019. *Nucleic Acids Research* 47**,** W636-W641. 10.1093/nar/gkz268

O'leary, N.A., Wright, M.W., Brister, J.R., Ciufo, S., Haddad, D., Mcveigh, R., et al. (2016). Reference sequence (refseq) database at ncbi: Current status, taxonomic expansion, and functional annotation. *Nucleic Acids Research* 44**,** D733-D745. 10.1093/nar/gkv1189

Rath, S., Heidrich, B., Pieper, D.H., and Vital, M. (2017). Uncovering the trimethylamine-producing bacteria of the human gut microbiota. *Microbiome* 5. 10.1186/s40168-017-0271-9

Rohland, N., and Reich, D. (2012). Cost-effective, high-throughput DNA sequencing libraries for multiplexed target capture. *Genome Research* 22**,** 939-946. 10.1101/gr.128124.111

Sabree, Z.L., Kambhampati, S., and Moran, N.A. (2009). Nitrogen recycling and nutritional provisioning by blattabacterium, the cockroach endosymbiont. *Proceedings of the National Academy of Sciences* 106**,** 19521-19526. 10.1073/pnas.0907504106

Stewart, F.J., Ottesen, E.A., and Delong, E.F. (2010). Development and quantitative analyses of a universal rrna-subtraction protocol for microbial metatranscriptomics. *The ISME Journal* 4**,** 896-907. 10.1038/ismej.2010.18

Tinker, K.A., and Ottesen, E.A. (2016). The core gut microbiome of the american cockroach, *periplaneta americana*, is stable and resilient to dietary shifts. *Appl Environ Microbiol* 82**,** 6603-6610. 10.1128/AEM.01837-16

Vera-Ponce De León, A., Jahnes, B.C., Duan, J., Camuy-Vélez, L.A., and Sabree, Z.L. (2020). Cultivable, host-specific bacteroidetes symbionts exhibit diverse polysaccharolytic strategies. *Applied and Environmental Microbiology* 86. 10.1128/aem.00091-20

Wirth, J.S., and Bush, E.C. (2023). Automating microbial taxonomy workflows with phantasm: Phylogenomic analyses for the taxonomy and systematics of microbes. *Nucleic Acids Research* 51, 3067-3077. 10.1093/nar/gkad196
